# Towards Understanding the Bioactive Potential of Two Intriguing *Nepeta* Species: Metabolomic and Phylogenetic Perspectives

**DOI:** 10.3390/plants15121804

**Published:** 2026-06-11

**Authors:** Uroš Gašić, Tijana Banjanac, Luka Petrović, Jovana Petrović, Ladislav Luc, Branislav Šiler, Danijela Mišić, Milica Milutinović

**Affiliations:** Department of Plant Physiology, Institute for Biological Research “Siniša Stanković”—National Institute of the Republic of Serbia, University of Belgrade, Bulevar despota Stefana 142, 11108 Belgrade, Serbia; tbanjanac@ibiss.bg.ac.rs (T.B.); luka.petrovic@ibiss.bg.ac.rs (L.P.); jovana0303@ibiss.bg.ac.rs (J.P.); ladislav.luc@ibiss.bg.ac.rs (L.L.); branislav.siler@ibiss.bg.ac.rs (B.Š.); dmisic@ibiss.bg.ac.rs (D.M.)

**Keywords:** *Nepeta govaniana*, *Nepeta subsessilis*, GC-MS, HPLC-Orbitrap MS, plastid DNA, antioxidants, antimicrobial activity

## Abstract

*Nepeta govaniana* and *Nepeta subsessilis* display metabolomes typical for the genus *Nepeta* L. (Lamiaceae), predominated by monoterpenoid iridoids and phenolic acids. Underexplored phytochemical composition and largely undefined biological potential are the main reasons for the underutilized status of these two intriguing species. This study fills some of the existing knowledge gaps by comprehensively analyzing the composition of methanol-soluble nonpolar, semi-polar, and polar metabolites in leaves, and providing the information about antimicrobial and antioxidant potential. Integration of comprehensive HPLC/MS and GC/MS metabolomics with plastid loci-derived (trnL-F, rbcL, and matK) phylogenetic data, revealed the phylogenetic relatedness of *N. govaniana* and *N. subsessilis* with congeneric species, and placed them within the *Nepeta*’s chemotype A whose members produce both iridoid aglycones and glycosylated iridoids. Methanol extracts of these two phylogenetically related species displayed a notable antioxidant potential, but were less efficient as antimicrobial agents. Such results draw from the predominance of methanol-soluble polar compounds (polyphenolics and iridoid glycosides), exhibiting respectable antioxidant potential, and lower abundance of nepetalactone-type iridoids, known as potent antimicrobials. *N. govaniana* is here highlighted as a slightly more efficient antimicrobial and antioxidant agent than *N. subsessilis*, which can be ascribed to a higher content of methanol-soluble metabolites in leaves.

## 1. Introduction

The genus *Nepeta* (catmints, catnips) comprises 296 accepted species with a native range from temperate Eurasia to Macaronesia and tropical East Africa [1]. These plants are known for the production of valuable specialized metabolites, belonging predominantly to terpenes and polyphenolics. The insignia of the genus are monoterpenoid iridoids nepetalactones that are absent in the remaining genera of the subfamily Nepetoideae (family Lamiaceae). These metabolites are attributed to numerous biological activities, including repellency against a vast array of arthropods and behavioral effects on cats [2,3,4,5,6,7]. Some *Nepeta* species are widely distributed and commercially exploited in the pharmaceutical and pet-toy industries or as horticultural plants, while many representatives of the genus with either a restricted geographical range or an endemic status remain underutilized. Previous phytochemical and pharmaceutical studies considered only a portion of the extant *Nepeta* taxa, with fragmentary insights into their metabolomes and bioactivities. Moreover, intra-generic phylogenetic relations in catmints remain largely unresolved, making it difficult to interpret chemical diversity in a phylogenetic context, which was recently demonstrated as an efficient approach to perform unambiguous chemotype assignment [8], and possibly predict the species’ bioactive properties.

With the aim to contribute in filling the knowledge gaps, this study was focused towards two underexplored but intriguing *Nepeta* species, *N. govaniana* (Wall. ex Benth.) Benth. and *N. subsessilis* Maxim. *N. govaniana*, known as Himalayan catmint, is native to Pakistan, Nepal, and the western Himalayas [1], and is well adapted to high-altitude harsh environments. *N. subsessilis*, commonly named short-stalked catmint, is native to Japan [1], but has been widely used as an ornamental plant due to its huge lavender-blue flowers, pleasant odor, and suitability for temperate climates and less challenging environments. Previous phytochemical studies on *N. govaniana* and *N. subsessilis* were focused primarily on the constituents of essential oils (EOs) [9,10,11,12,13,14], but polar metabolite profiles that would cover glycosylated iridoids and polyphenolics are mainly lacking. Both species are generally recognized as minor producers of iridoid aglycones of the nepetalactone type. The main constituents of *N. govaniana* EOs are reported to be pregeijerene [9,10,11,13], geijerene [9,13], *trans,cis*-iridolactone [9], *cis,trans*- and *cis,cis*-nepetalactone [9,10,12], germacrene D [12], *β*-elemene [12], and isoiridomyrmecine [10]. On the other hand, *β*-caryophyllene, *α*-pinene, and caryophyllene oxide were previously identified as major constituents of *N. subsessilis* EOs, while *cis,cis*- and *trans,cis*-nepetalactone were present as minor constituents [14]. Studies related to biological activities of the two *Nepeta* species are scarce and limited to the antibacterial [11] and cytotoxic effects [13] of *N. govaniana* and the larvicidal activity of *N. subsessilis* against *Aedes aegypti* [14].

We here aimed at comparatively analyzing chemical profiles, antimicrobial activities, and enzymatic and non-enzymatic components of the antioxidant mechanisms of *N. govaniana* and *N. subsessilis.* This study expands the knowledge of chemodiversity within the genus *Nepeta* and, for the first time, provides a comprehensive characterization of the iridoid composition of these two spesies through the simultaneous analysis of simple nepeta-type iridoid aglycones and glycosylated iridoids using state-of-the-art analytical approaches. In addition, a multidimensional phytochemical dataset acquired by the parallel profiling of major metabolite classes (terpenes, polyphenolics, fatty acids, and phenylethanoids) offers a more holistic understanding of the metabolomes of these species’ chemistry than is typically available for *Nepeta* taxa. By growing the plants under controlled greenhouse conditions, we intended to exclude possible metabolic differences originating from varying environmental factors towards most exact profiling of the specialized compounds they produce. The obtained results were anticipated to be juxtaposed to the phylogenetic positions of both species within the genus *Nepeta* obtained using three plastid markers from the plant DNA barcoding system. This integrated approach was aimed at providing an insight into the constitutive adaptations at the genetic, metabolic, and proteomic levels, thereby contributing to a deeper understanding of the inherent biochemical diversity, phylogeny, and chemical evolution within the genus *Nepeta*. The work therefore contributes to the emerging field of evolutionary phytochemistry, where secondary metabolites are examined as phylogenetically informative traits rather than merely bioactive constituents.

## 2. Results and Discussion

### 2.1. Untargeted Metabolomics of N. govaniana and N. subsessilis Leaves Using HPLC/Orbitrap MS

Untargeted profiling of leaf methanol extracts of Himalayan catmint (*N. govaniana*) and short-stalked catmint (*N. subsessilis*) led to the identification of totally 145 compounds which are presented within the Table 1. The same table also contains the MS data and a list of references confirming the presence of the given compound either in the genus Nepeta or in the Lamiaceae family.

**Figure 1:**
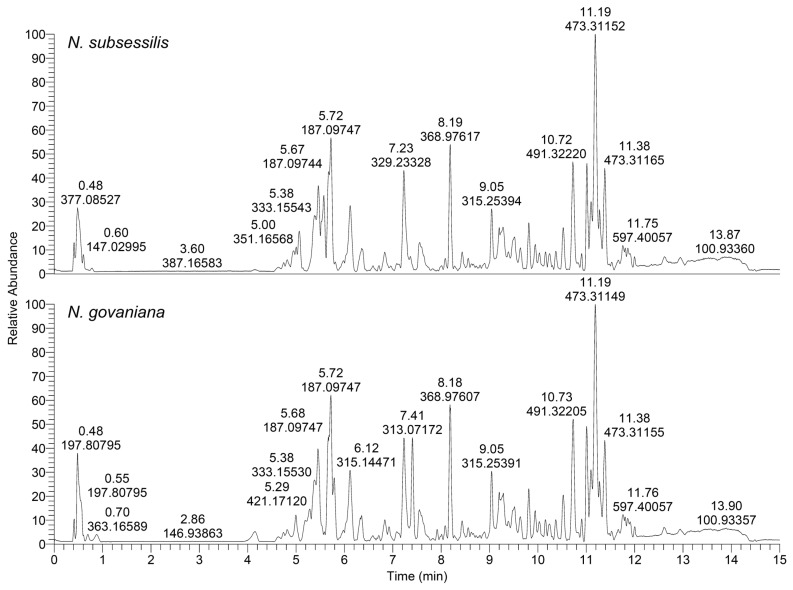
Representative HPLC/Orbitrap MS base peak chromatograms of *N. subsessilis* and *N. govaniana* methanol extracts.

The identified compounds were divided into nine groups according to their chemical structures: (1) hydroxybenzoic acid derivatives (14 compounds), (2) hydroxycinnamic acid derivatives (47 compounds), (3) phenylethanoids (8 compounds), (4) iridoid glycosides (10 compounds), (5) iridoid aglycones (10 compounds), (6) flavonoid glycosides (24 compounds), (7) flavonoid aglycones (10 compounds), (8) fatty acids (7), and (9) other metabolites (15 compounds). Representative HPLC/Orbitrap MS total ion chromatograms (TICs) of *N. subsessilis* and *N. govaniana* methanol extracts are presented in Figure 1. Table S1 lists peak areas of all identified compounds.

The class of polyphenolics was represented with totally 95 compounds (Table 1). Of the 14 derivatives of hydroxybenzoic acids, only 1 was free acid—hydroxybenzoic acid (**6**), while the other 13 were found in a glycosylated form, with MS^2^ fragmentation patterns displaying a characteristic loss of either pentosyl or hexosyl moieties (−132 Da and −162 Da, respectively). Some of the glycosides were also esters with either syringic acid (**10** and **12**) or glutaric acid (**8**). Regarding hydroxycinnamic acids, besides derivatives in the form of glycosides and esters (with tartaric, quinic, and shikimic acid), the majority of the identified compounds were unique derivatives of caffeic acid (**22**), known to be common for the Lamiaceae family [87,88]. Two caffeic acid oligomers were recorded in *N. govaniana*, yunnaneic acids D and I (**27** and **36**, respectively), and these compounds were previously isolated from the roots of *Salvia yunnanensis* [89,90]. Rosmarinic acid (**46**) was previously recognized as the most abundant phenolic acid in *Nepeta* species [88], and it was also abundant in the leaves of *N. govaniana* and *N. subsessilis*. Derivatives of rosmarinic acid, including methyl rosmarinate (**57**), salvianic acid A (**47**) and its hexoside (**17**), salvianolic acids B and C (**38** and **59**, respectively), sagerinic acid (**48**), and salviaflaside (**32**) were abundant in both *N. govaniana* and *N. subsessilis* (Table 1 and Table S1). Methyl rosmarinate (**57**) was previously recorded in the leaves of numerous *Nepeta* species, including *N. angustifolia* [91], *N. asterotricha* [55], *N. glutinosa* [92], *N. prattii* [93], and *N. deflersiana* [94]. Salvianic acid A (also known as danshensu) was reported as the main bioactive component of *Salvia miltiorrhiza* [95], and was here found in *N. govaniana*. The two isomers of chlorogenic acid (**18** and **19**) were recorded only in *N. govaniana* leaves. Some hydroxycinnamic acid derivatives were present as glycosides (**49**, **50**) and esters (**33**, **43**, **44**, **51**, **53**), and they were especially abundant in the leaves of *N. govaniana*.

Totally 34 flavonoid compounds were identified in the leaves of *N. subsessilis* and *N. govaniana*, 24 out of which were found in the glycosylated form and 10 as aglycones. Simple flavonoids are represented by nine flavones (**114**, **115**, **116**, **118**, **119**, **120**, **121**, **122**, and **123**) and one flavanone, naringenin (**117**). Flavones were reported to be the most abundant group of flavonoids in *Nepeta*, particularly apigenin and luteolin glycosides and their methyl derivatives [16,77,88,96,97]. Not surprisingly, apigenin and luteolin, and their derivatives, predominated in *N. subsessilis* and *N. govaniana* leaves. Baicalein (**114**) and thymusin (**115**) were identified only in the leaves of *N. subsessilis*, but isothymusin (**119**) was recorded in both analyzed species. Thymusin and isothymusin were previously reported in *N. asterotricha* [55], the former being also found in *N. nuda* [18,98]. Cirsimaritin (**120**) was previously reported for several *Nepeta* species [16,18,77,98]. Chrysoeriol (**121**) was previously identified in *N. nuda* [18], while acacetin (**123**) was recorded in *N. nuda* [18,98], *N. rtanjensis,* and *N. argolica* [96], as well as in several *Nepeta* species (*N. cataria*, *N. ernesti-mayeri*, *N. faassenii*, *N. grandiflora*, *N. racemosa*, *N. nervosa*, *N. nuda*, *N. parnassica*, *N. rtanjensis*, *N. sibirica*, *N. argolica*, and *N. laevigata*) described by Mišić et al. [88]. Naringenin was previously recorded in many *Nepeta* species [18,88,98]. Leaves of *N. subsessilis* and *N. govaniana* were rich in flavone, flavanone, and flavonol glycosides. Among flavone glycosides, various derivatives of apigenin (**90**, **92**, **99**, **101**, and **110**), luteolin (**91**, **94**, **98**, **103**, **106**, and **112**), chrysoeriol (**100**, **111**, and **113**), and thymusin (**105** and **109**) were recorded. Apigenin 7-*O*-hexosides were reported to be abundant across *Nepeta* species [18,99,100]. Glycosides of flavonols were represented by derivatives of quercetin (**93**, **95**, and **96**) and kaempferol (**97** and **102**), and they were generally more abundant in *N. govaniana*. One glycoside of flavanone, hesperetin (**104**), was recorded only in *N. govaniana*. A wide diversity of flavonoid glycosides in the form of hexuronide conjugates were recorded in the analyzed samples of *N. govaniana* and *N. subsessilis*, including those aclylated with caffeoyl (**106**, **110**) and feruloyl moieties (**112**). Acetylated glycosides of thymusin (**109**) and chrysoeriol (**113**) were recorded only in *N. govaniana*. Flavones (mainly apigenin and luteolin derivatives) glycosylated with hexuronic (glucuronic) acid, often with another acyl residue (acetyl, coumaroyl, caffeoyl or similar), have been previously reported as constituents of several *Nepeta* species, including *N. curviflora* [101], *N. nuda* [18,98,102], *N. rtanjensis,* and *N. argolica* [96].

Regarding phenylethanoids, eight compounds were identified, and their qualitative content differed in leaves of *N. subsessilis* and *N. govaniana*. Leaves of *N. govaniana* contained verbascoside (syn. acteoside) (**64**) and its derivative diacetyl-verbascoside (**66**), as well as kankanoside G (**67**), none of which were recorded in *N. subsessilis*. Teupolioside (**62**), dehydroacteoside (**63**), 3‴O-methylcrenatoside (**65**), cuneataside D (**68**), and premnethanoside A or B (**69**) were recorded exclusively in the leaves of *N. subsessilis*. Verbascoside was previously found in *N. ucrainica* [48] and *N. cataria* [103] and teupolioside was previously reported in *N. cataria* [103]. To the best of our knowledge, other phenylethanoids identified in this study were not previously found in any *Nepeta* species.

Totally 23 iridoid metabolites were recorded in the analyzed *N. subsessilis* and *N. govaniana* samples, 13 in the form of aglycones and 10 glycosylated compounds. Six lactone-type iridoid aglycones were recorded: two stereoisomers of nepetalactone (**88**, **89**), two isomers of dihydronepetalactone (**83** and **85**), 5,9-dehydronepetalactone (**87**), and nepetalic acid (**86**). Previous phytochemical studies of *N. govaniana* describe *cis*,*trans*- and *cis*,*cis*-nepetalactone isomers [9,10,12], although in variable amounts and ratios. However, two other studies reported no nepetalactones in *N. govaniana* [13,104]. Previous studies reported the presence of *cis*,*trans*- and *trans*,*cis*-nepetalactone as minor components of *N. subsessilis* essential oils [14]. These two stereoisomers of nepetalactone were clearly chromatographically separated and identified in *N. subsessilis* and *N. govaniana* samples based on their exact masses determined from MS and MS^2^ spectra. The assignment of the two 7S type nepetalactones—*cis*,*trans*- and *cis*,*cis*-nepetalactone—was unambiguously confirmed based on the MS data of the authentic standards. Within this study, two isomers of dihydronepetalactone (**83** and **85**) were recorded in *N. govaniana* samples, while only dihydronepetalactone 1 (**83**) was identified in leaf samples of *N. subsessilis*. 5,9-Dehydronepetalactone (**87**) was here recorded in both *N. govaniana* and *N. subsessilis*, and this compound was often considered a degradation product of nepetalactone formed during the extraction process [8,105]. This compound was previously reported in *N. cataria* [8,105], *N. nuda* [8,98], *N. rtanjensis* [8,88,96,97], *N. sibirica* [8,106], *N. ernesti-mayeri*, *N. grandiflora*, *N. laevigata, N. stewartiana*, and *N. parnassica* [8]. Nepetalic acid (**86**), another degradation product of nepetalactone, was abundant in the leaves of both analyzed species. Nepetalic acid was previously recorded in numerous *Nepeta* species, including *N. cataria*, *N. nuda*, *N. ernesti-mayeri*, *N. grandiflora*, *N. laevigata*, *N. rtanjensis*, *N. sibirica*, *N. stewartiana*, *N. parnassica* [8,98,105]. To the best of our knowledge, derivatives of nepetalactone, compounds **83**, **85**, **86**, and **87**, were not previously reported for *N. govaniana* and *N. subsessilis*. Aglycones of lactol-type iridoids were represented by nepetalactol (**82**) and compounds structurally resembling the aglycone parts of iridoid glycosides, such as deoxyloganetic acid (**80**), nepetaracemoside B aglycone (**81**), and deoxygeniposide aglycone (**84**). Nepetalactol (**82**), considered to be the common precursor of all iridoids in plants, was recorded in both *N. subsessilis* and *N. govaniana*. Deoxyloganetic acid (**80**), a proposed precursor in the biosynthesis of 1,5,9-*epi*-deoxyloganic acid (**75**) in *Nepeta* species [8], was recorded only in the samples of *N. govaniana* leaves. Nepetaracemoside B aglycone (**81**) and deoxygeniposide aglycone (**84**) most likely arise through the deglycosylation of nepetaracemoside B and deoxygeniposide, respectively. Compound **81** was here recorded only in *N. govaniana* samples, and it was previously found in *N. cataria*, *N. nuda*, *N. rtanjensis*, *N. sibirica*, *N. ernesti-mayeri*, *N. grandiflora*, *N. laevigata*, *N. stewartiana*, and *N. parnassica* [8]. Deoxygeniposide aglycone (**84**) was identified in both analyzed *Nepeta* species, and was previously reported in *N. rtanjensis*, *N. sibirica*, *N. parnassica*, *N. nuda*, and *N. stewartiana* [8].

To the best of our knowledge, iridoid glycosides were not previously recorded in *N. govaniana* and *N. subsessilis*, and here we found 10 compounds from this group. Among iridoid glycosides of the 11-COOH type, 1,5,9-*epi*-deoxyloganic acid (**75**) predominated, although nepetanudoside (**72**), 11-*O*-hexosyl-1,5,9-*epi*-deoxyloganic acid (**70**), and 11-*O*-pentosyl-1,5,9-*epi*-deoxyloganic acid (**74**) were also identified. Compound **70** was previously recorded for *Nepeta* species, including *N. cataria*, *N. nuda*, *N. rtanjensis*, *N. sibirica*, *N. ernesti-mayeri*, *N. grandiflora*, *N. laevigata, N. stewartiana*, *N. argolica*, and *N. parnassica* [8,96,97,98,107]. On the other hand, compound **74** was reported for *N. cataria*, *N. nuda*, *N. rtanjensis*, *N. ernesti-mayeri*, *N. grandiflora*, *N. laevigata, N. argolica*, and *N. parnassica* [8,96]. The 11-CH_3_ type IGs were represented by 5-deoxylamiol (**76**), nepetaracemoside A (**77**), nepetaside (**78**), and nepetariaside (**79**). Nepetaside (**78**) and nepetariaside (**79**) were identified in both *N. govaniana* and *N. subsessilis*, and were previously reported for *N. cataria*, *N. nuda*, *N. rtanjensis*, *N. ernesti-mayeri*, *N. grandiflora*, *N. laevigata, N. stewartiana*, *N. argolica*, and *N. parnassica* [8]. On the other hand, 5-deoxylamiol (**76**) was recorded only in *N. subsessilis*, while nepetaracemoside A (**77**) was present in *N. govaniana*. 5-Deoxylamiol (**76**) was previously reported in *N. sibirica* and *N. ernesti-mayeri* [8]. The presence of nepetaracemoside A (**77**) was previously confirmed for *N. mussinii* (syn. *N. racemosa*), *N. cataria*, *N. nuda*, *N. rtanjensis*, *N. ernesti-mayeri*, *N. grandiflora*, *N. laevigata, N. stewartiana*, *N. argolica*, and *N. parnassica* [8,58]. Two more IGs not assigned to any of the above subgroups were recorded, adenosmoside (**71**) and 6α-hydroxyadoxoside (**73**). Compound **71** was previously recorded in *N. parnassica* and *N. nuda* [8], while 6α-hydroxyadoxoside (**73**) was found in *N. nuda* [98].

Fatty acids were present in methanol extracts of *N. govaniana* and *N. subsessilis* leaves, in either oxidized (**128**) or hydroxylated forms (**124**, **125**, **126**, **127**, **129**, and **130**). The literature data on the fatty acid composition of the genus *Nepeta* are rather scarce and are limited to seeds, where saturated (palmitic, oleic, stearic acid) and polyunsaturated fatty acids (linoleic, linolenic acid) were reported [78,108].

HCA was performed on the semi-quantitative HPLC/Orbitrap MS data (peak areas), adopting the Pearson’s method for cluster agglomeration (Figure S1). The HCA plot clearly distinguishes the two analyzed species based on the composition of methanol-soluble metabolites in leaves.

### 2.2. Untargeted GC/MS Metabolomics of N. govaniana and N. subsessilis Leaves

**Figure 2:**
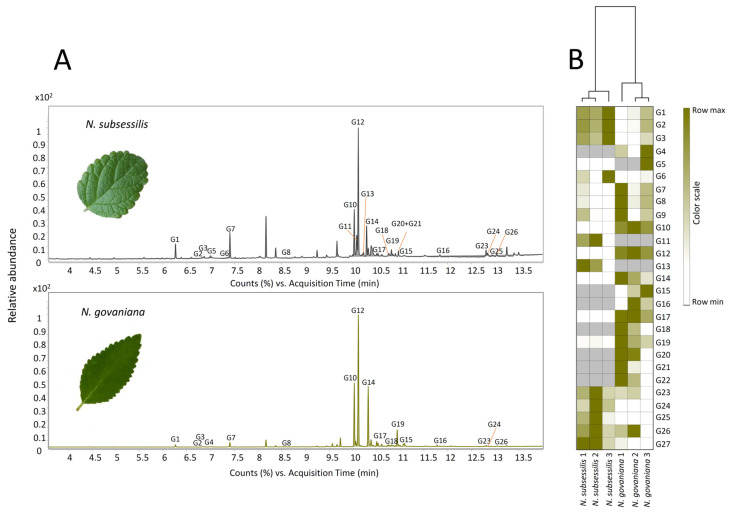
GC/MS profiling of semi- and nonpolar metabolites in methanol extracts of *N. subsessilis* and *N. govaniana* leaves. (**A**) Representative total ion chromatograms (TICs). (**B**) Heat map of the semi-quantitative data (peak areas) of the 27 identified volatile organic compounds (VOCs), with both samples and metabolites arranged according to the hierarchical cluster analysis (HCA) adopting the Pearson’s method of cluster agglomeration. Values are scaled between minimum and maximum, for each row independently, as indicated by the color scale. Metabolites are labeled with numbers, corresponding to those presented in Table 2.

Qualitative GC/MS profiling of the volatile organic compounds (VOCs) present in methanol extracts of *N. govaniana* and *N. subsessilis* leaves was performed, and representative TIC GC/MS chromatograms are shown in Figure 2A.

In total, 27 terpenes were identified (Table 2), including monoterpenes (6 monoterpene hydrocarbons and 10 oxygenated monoterpenes), sesquiterpenes (5 sesquiterpene hydrocarbons and 1 oxygenated sesquiterpene), and diterpenes (4 diterpenes and 1 oxygenated diterpene).

The qualitative composition of monoterpene hydrocarbons was highly conserved across the analyzed taxa, with *α*-pinene (**G1**), *β*-pinene (**G3**), sabinene (**G2**), and D-limonene (**G6**) being present in both *N. govaniana* and *N. subsessilis.* o-Cymene (**G5**) was recorded only in *N. govaniana*, while *β*-myrcene (**G4**) was recorded only in the leaves of *N. subsessilis*. Oxygenated monoterpenes were highly abundant in the analyzed samples, and this group of metabolites was represented with 1,8-cineole (**G7**), *δ*-terpineol (**G8**), pulegone (**G9**), five stereoisomers of nepetalactone (**G10**–**G14**) and nepetalactone derivatives, 5,9-dehydronepetalactone (**G15**) and dihydronepetalactone (**G16**). In natural sources, nepetalactones usually occur in the form of four 7S stereoisomers, namely 7S-*trans*,*trans*-, 7S-*cis*,*trans*-, 7S-*trans*,*cis*-, and 7S-*cis*,*cis*-nepetalactone. According to the IUPAC nomenclature, 7S refers to the configuration at C7, the first part of the name refers to the configuration at the ring junction (4a-7a) and the second part to the configuration of the 7a-7 bond [109].

Only rarely, nepetalactone enantiomers of the 7R- configuration were identified in *Nepeta* species, including *N. elliptica* [11,110,111], *N. parnassica* [112], and *N. nuda* [113,114]. In this study, four nepetalactone stereoisomers of the 7S type—*trans*,*trans*- (**G10**), *cis*,*trans*- (**G12**), *trans*,*cis*- (**G13**), and *cis*,*cis*-nepetalactone (**G14**)—were recorded in *N. govaniana* samples, and their identification was additionally confirmed using both authentic standards and the literature data [8,88,96]. Interestingly, one more isomer of nepetalactone was found in the *N. subsessilis* leaves, which, most likely, resembles the 7R configuration (**G11**). However, due to the technical limitations of the analytical instruments employed, we were not able to unambiguously confirm the stereochemistry of the **G11** nepetalactone. Using the GC/MS approach, 5,9-dehydronepetalactone (**G15**) and one isomer of dihydronepetalactone (**G16**) were recorded only in the *N. subsessilis* leaves. Obviously, iridoid profiling of nepetalactones adopting the two different analytical approaches (HPLC/Orbitrap MS and GC/MS) revealed contrasting results.

It is not surprising that more nepetalactone isomers were recorded using the GC/MS instrument, since it is more sensitive to volatile compounds, such as nepetalactones. On the other hand, HPLC/Orbitrap MS technique is more suitable for the analysis of polar compounds, including iridoid glycosides, which represent a significant portion of the overall iridoid content. Therefore, it was essential to combine the two analytical approaches stemming from different physicochemical principles to capture the iridoid diversity at its maximum. Such a comprehensive metabolomics approach, previously developed by Banjanac et al. [8], was efficient in distinguishing the three *Nepeta* chemotypes based on the presence/absence of nepetalactone (NL)-type iridoid aglycones (IAs) and iridoid glycosides (IGs). The cited study suggests the existence of *Nepeta* taxa producing both NL-type IAs and IGs (referred to as chemotype A), taxa producing only IGs (chemotype B), and those producing neither NL-type IAs nor IGs (chemotype C). This diversity of iridoids was, at least partially, attributed to evolutionary gains and losses of key biosynthetic genes [8]. Based on this study, *N. subsessilis* and *N. govaniana* can be classified as chemotype A, as they produce both NL-type IAs and IGs. Furthermore, they most likely belong to the same subchemotype (A2), as they dominantly produce the same nepetalactone stereoisomer in leaves, *cis*,*trans*-nepetalactone. It was reported that *N. sibirica*, *N. cataria*, *N. nuda*, and *N. ernesti-mayeri* also belong to subchemotype A2 [8].

As for sesquiterpenes, *β*-caryophyllene (**G17**) and germacrene D (**G19**) were highly abundant in *N. govaniana* and *N. subsessilis* leaves. These compounds are generally often recorded in leaf methanol extracts [8,102] and EOs of various *Nepeta* species [64,98,115,116]. Sesquiterpenes were more abundant in *N. subsessilis*, and humulene, *γ*-himachalene, bicyclogermacrene, and humulene epoxide I were recorded only in the leaves of this species (Table 2). Totally five diterpenes were recorded. Three isomers of a neophytadiene, 2-phyten, and an oxygenated diterpene phytol were found in all analyzed samples. These diterpenes were also found to be abundant in methanol extracts of other *Nepeta* species [8,98]. HCA based on relative GC/MS data and using Pearson’s algorithm of cluster agglomeration clearly separated the two *Nepeta* taxa (Figure 2B). Raw GC/MS data, including areas of peaks assigned to specific volatile terpenes in this study, are presented in Table S2.

### 2.3. Phylogenetic Positioning of N. govaniana and N. subsessilis Within the Genus Nepeta

**Figure 3:**
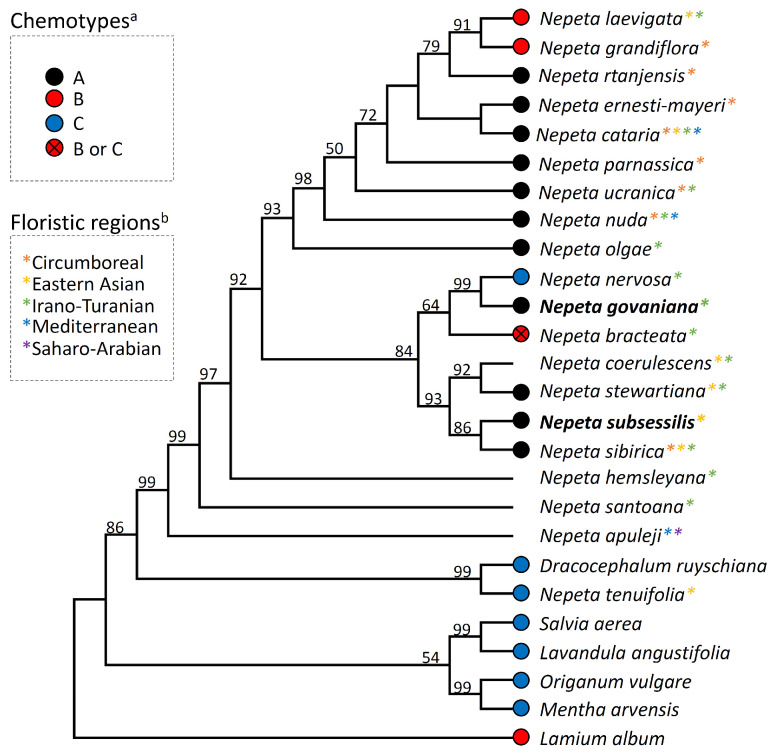
Phylogenetic relations of *N. govaniana* and *N. subsessilis* with 18 congeneric species, as revealed by three plastid DNA markers (*trn*L-F, *rbc*L, and *mat*K). Publicly available sequences belonging to the iridoid non-producing representatives of the subfamily Nepetoideae (*Dracocephalum ruyschiana*, *Salvia sclarea*, *Lavandula angustifolia*, *Origanum vulgare*, and *Mentha arvensis*), and one iridoid-glycoside producing representative of the subfamily Lamioideae (*Lamium album*) were also included in the analysis as outgroups. Accession numbers are presented in Table S3. The constructed phylogenetic tree was used as a framework to map the geographic distribution and iridoid variability of the analyzed *Nepeta* species. The chemotypes were determined according to ^a^ Banjanac et al. [8], and the postulated criterion was whether a specific species produces both IAs and IGs (chemotype A, black circles), only IGs (chemotype B, red circles), or they completely lack iridoids (chemotype C, blue circle). It was impossible to unambiguously assign *N. bracteata* to either chemotype because this species was reported to lack nepetalactone-type iridoids, but has never been analyzed for the content of IGs (crossed red circle). Floristic regions were proposed by ^b^ Takhtadzhian et al. [123] and are represented by different colors of the superscript star symbols, as explained in the symbol legend.

The literature data lack information on the phylogenetic position of *N. subsessilis* and *N. govaniana* within the genus *Nepeta*. The most comprehensive phylogenetic studies were those employing the genomic ITS sequences [8,117,118], which gave a good starting point towards elucidating the overall phylogeny of the genus. Here, we reconstructed the phylogenetic relations with other congeneric species using *trn*L-F, *rbc*L, and *mat*K plastid markers belonging to the plant DNA barcoding system (Figure 3).

Plastid DNA provides valuable information about the mode of organelle inheritance in plants. Based on the results obtained with *N. cataria* [119], it could be presumed that biparental inheritance with egg cell-derived organelle predominating those from male gametes in zygote might be the common inheritance mechanism in catmints. However, other transmission modes cannot be excluded without further experimental evidence. The accelerating number of plastid genome sequences in public databases is expected to provide tools to resolve these complex issues in the future.

Two previous studies employed the same combination of plastid loci for the construction of the genus *Nepeta* phylogenetic tree [8,120], and these publicly available sequences were employed here. A survey of the literature reveals several publications where different DNA barcoding markers were used (e.g., [18,118,121,122]), but they cannot be used for comparison with our data. However, an increasing number of publicly available chloroplast genomes of *Nepeta* species enabled us to search for the sequences of *trn*L-F, *rbc*L, and *mat*K loci for additional seven *Nepeta* species and to include them in the analysis towards increasing the discrimination resolution (Table S3). Thus, the obtained phylogenetic tree comprised totally 20 *Nepeta* species and 6 species belonging to other genera of the Lamiaceae family as outgroups (Figure 3 and Table S3).

The dendrogram presented in Figure 3 shows close relatedness of *N. govaniana* with *N. nervosa* and *N. bracteata*, while *N. subsessilis* was clustered closely with *N. stewartiana*, *N. thomsonii*, and *N. sibirica*. These seven species formed a coherent clade, clearly distinguished from the remaining *Nepeta* species selected for this study. This is consistent with recent studies by Rose et al. [118] and Banjanac et al. [8], revealing a close relatedness of *N. nervosa*, *N. sibirica*, and *N. stewartiana*. Phylogenetic relatedness reconstructed here was further supported by the study of Chen et al. [124], who positioned *N. stewartiana* close to *N. bracteata*, *N. hemsleyana*, and *N. tenuifolia*. However, some preceding studies have placed *N. sibirica* within the section *Macronepeta*, while *N. nervosa* was assigned to the section *Spicata* [125]. Similarly, Jamzad et al. [117] placed *N. sibirica* and *N. nervosa* in separate clades, namely IIA and IIB, respectively. Phylogenetic relationships of other *Nepeta* species analyzed within the present study resembled, to a large extent, those previously proposed by adopting ITS regions and/or plastid loci [8,118]. Balkan endemics *N. rtanjensis*, *N. parnassica*, and *N. ernesti*-*mayeri*, belonging to the *N. sibthorpii* complex of the section *Nepeta* [125], clustered closely in this study. They were also closely grouped with *N. cataria*, *N. ucranica*, *N. nuda*, and *N. olgae*, which follows the phylogenetic relationships suggested by Banjanac et al. [8]. Similar to the study by Banjanac et al. [8], *N. grandiflora* and *N. laevigata* were found to be closely related. A high level of similarity between the phylogenetic trees constructed based on the genomic ITS- [8,117,118] and plastid DNA-derived trees in the present study may draw from the similarities in the inheritance mode—biparental transmission of both plastid and nuclear genomes, which generates indistinct phylogenetic signals. However, such a hypothesis requires further experimental confirmation.

The constructed phylogenetic tree was further used as a framework to map the geographical distribution of the set of analyzed *Nepeta* species (Figure 3) and to interpret phylogeographic patterns among them. *N. govaniana* formed a well-supported clade with *N. nervosa* and *N. bracteata*, all belonging to the Irano-Turanian floristic region [123], characterized by a predominantly continental climate. Although being geographically isolated in Japan, which belongs to the Eastern Asian region, *N. subsessilis* segregates closely with *N. sibirica* that has rather wide distribution within the Eastern Asian, Irano-Turanian, and Circumboreal floristic regions. These two species form a well-supported clade with *N. stewartiana* and *N. coerulescens*, with native ranges within the Eastern Asian and Irano-Turanian floristic regions*. N. cataria* and *N. nuda* overlap in their geographic distribution within the Circumboreal, Irano-Turanian, and Mediterranean regions, although *N. cataria* can also be found in the Eastern Asian region. Balkan endemics *N. rtanjensis*, *N. ernesti-mayeri*, and *N. parnassica* represent isolated entities within the Circumboreal region. Interestingly, two species belonging to the chemotype B (IG-producing species), *N. grandiflora* and *N. laevigata,* are geographically well distinguished, with *N. grandiflora* inhabiting the temperate Circumboreal region, while the range of *N. laevigata* distribution is within the Eastern Asian and Irano-Turanian floristic regions.

Following the strategy proposed by Banjanac et al. [8], iridoid diversity was projected onto the phylogenetic tree constructed based on the plastid loci (Figure 3), although metabolomics data for some of the species (*N. thomsonii*, *N. hemsleyana*, *N. santoana*, and *N. dentata*) were missing in the literature. This study for the first time describes the profiles of both iridoid aglycones (IAs) and glycosylated iridoids (IGs) in the leaves of *N. subsessilis* and *N. govaniana*. Although being phylogenetically closely related, these two *Nepeta* species display considerable differences in the qualitative and quantitative composition of iridoids. Integration of the metabolomic and phylogenetic data further strengthened the relatedness of *N. subsessilis* and *N. govaniana* with *N. sibirica*, and *N. stewartiana*, which were previously assigned to chemotype A, namely the producers of iridoids, in both forms, IAs and IGs [8]. On the other hand, the two species analyzed in this study are also phylogenetically close to *N. nervosa*, which is a nepetalactone non-producing species (chemotype C), lacking some of the key components of the iridoid biosynthetic machinery, namely genes coding for geraniol synthase (GES), iridoid synthase (ISY), and NAD-dependent nepetalactol-related short-chain dehydrogenase/reductase (NEPS) enzymes belonging to the subgroup of NEPS-cyclases [8]. This conforms to the hypothesis concerning the evolutionary convergence in the production of iridoids and their remarkable heterogeneity within the genus *Nepeta*. Interestingly, *N. nervosa*, *N. sibirica*, and *N. stewartiana* were found to share the unique iridoid biosynthesis features within the genus *Nepeta*, namely the prevalence of major latex protein-like (MLPL) enzymes-mediated cyclization of 8-oxocitronellyl enolate into nepetalactol over the NEPS-cyclase-mediated reactions [8]. This is, most likely, the case in the whole phylogenetic lineage, comprising also *N. govaniana* and *N. subsessilis*, although these assumptions require further experimental evidence.

### 2.4. Antioxidative Capacity of N. govaniana and N. subsessilis

**Figure 4:**
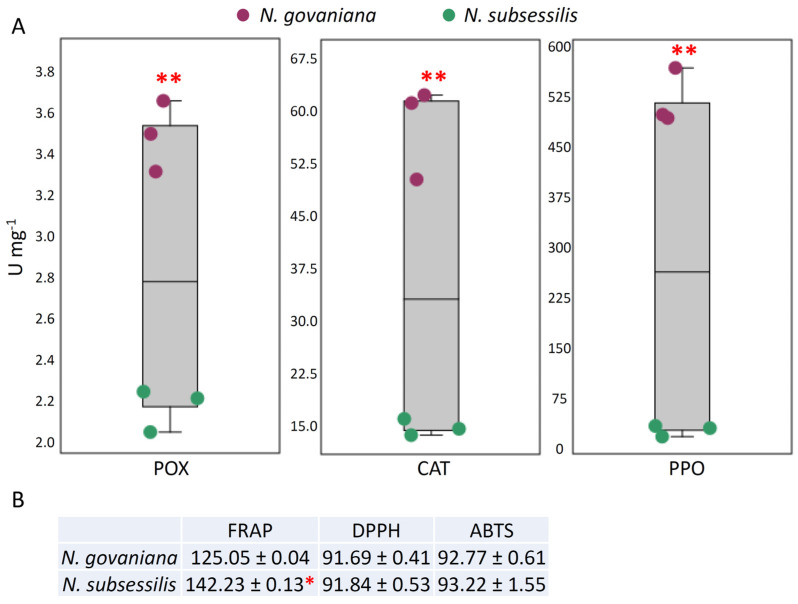
(**A**) Activities of antioxidant enzymes in *N. govaniana* and *N. subsessilis* leaves. Results are represented as U mg^−1^ of total proteins. (**B**) Antioxidant activity of methanol extracts of *N. govaniana* and *N. subsessilis* leaves, measured by FRAP, DPPH, and ABTS assays. Results are represented as mmol gallic acid equivalents (GAE) per 100 mg leaf FW. Asterisks denote statistically significant values * *p* < 0.05, ** *p* < 0.005 calculated using the Student’s *t* test. Abbreviations: POX—peroxidase; CAT—catalase; PPO—polyphenol oxidase.

Enzymatic antioxidative capacity of the *N. govaniana* and *N. subsessilis* methanol extracts was determined by the activities of peroxidase (POX), catalase (CAT), and polyphenol oxidase (PPO). POX and CAT are the key enzymes responsible for the removal of H_2_O_2_, while PPO, although not classified as a classical antioxidant enzyme, contributes indirectly to oxidative stress mitigation. PPO oxidizes phenolics into quinones that can stabilize reactive oxygen species (ROS), but can also activate various signaling pathways that play crucial roles in oxidative stress responses. *N. govaniana* exhibited much higher CAT and particularly PPO activity compared to *N. subsessilis* (Figure 4).

Although *N. govaniana* also exhibited higher POX activity compared to *N. subsessilis*, the activity of this enzyme was relatively low in both species. These results demonstrate that *N. govaniana* possess stronger enzymatic antioxidative defense mechanisms compared to *N. subsessilis*. This aligns with *N. govaniana* adaptation to high-mountain environments, with extreme environmental conditions where stronger constitutive antioxidative defense mechanisms, reflected in elevated CAT and PPO activity, can contribute to enhanced stress tolerance. Based on the immunoblot analysis, six isoforms of POX could be distinguished in the leaves of these two *Nepeta* species, with one isoform being unique to each species—isoform IV was present in *N. govaniana* only and isoform V in *N. subsessilis* (Figure S2). The most abundant isoforms in *N. govaniana* were isoforms 2, 4, 6 and 7, while in *N. subsessilis* isoforms 1, 6 and 7 prevailed. An increased total POX content is in accordance with an increment in the enzyme activity in *N. govaniana* (Figure 4A). On the other hand, the immunoblot analysis showed that only one isoform of either CAT or PPO proteins is detected in both species (Figure S2). An enhanced CAT protein content is in accordance with the increment in the enzyme activity of *N. govaniana* (Figure 4B). While PPO abundance was decreased in *N. govaniana,* it must be remembered that the immunoblot analysis gives indications about the synthesis of PPO subunit and does not necessarily reflect the enzyme activity.

A higher antioxidant capacity of *N. govaniana* than that of *N. subsessilis* may be attributed to higher quantities of polyphenolics and the presence of some of the compounds from this class exclusively in *N. govaniana*. The antioxidative capacity of these two species can be associated with the action of polyphenolics, including chlorogenic acid [126], coumarin derivatives [127], and flavonoids such as hesperetin [128] and chrysoeriol [129]. Additionally, quercetin [130] and kaempferol [131], which are more abundant in *N. govaniana*, as well as phenylethanoid verbascoside [132], may significantly contribute to the overall *N. govaniana* antioxidant capacity. As both species were exposed to the same environmental conditions, the observed differences reflect, most likely, inherent, constitutive adaptations rather than environment-induced responses. These findings suggest that *N. govaniana* has evolved a stronger basal enzymatic antioxidative defense at both the metabolic and enzymatic levels, whereas *N. subsessilis* exhibits a weaker constitutive antioxidative protection system. Considering non-enzymatic antioxidative capacity, the obtained results of the FRAP assay indicate that *N. subssesilis* possesses slightly stronger ferric-reduced antioxidative power compared to *N. govaniana*, although no significant differences were recorded in radical scavenging capacity in the DPPH and ABTS assays (Figure 4B). These results indicate that *N. subsessilis* possesses a slightly larger pool of electron-donating antioxidants, while the antioxidant defense of *N. govaniana* relies more on the enzymatic antioxidative mechanisms. These results are consistent with the previous studies. It has been shown that the methanol extracts of *N. rtanjensis*, *N. sibirica,* and *N. nervosa* possessed strong antioxidant activities as manifested in ABTS and DPPH assays, while the FRAP assay showed high ferric-reducing abilities for all three tested species. This strong antioxidant potential could be attributed to phenolic acids, and in the first place to rosmarinic acid [133].

### 2.5. Antimicrobial Activity of N. subsessilis and N. govaniana Methanol Extracts

There are no data in the literature concerning the antimicrobial activity of *N. subsessilis*. On the other hand, *N. govaniana* EO was reported to exhibit weaker antimicrobial activity towards pathogens such as *Escherichia coli*, *Pseudomonas aeruginosa*, *Staphylococcus aureus*, *Pasteurella multocida*, *Pseudomonas vulgaris*, *Serratia marcescens*, *Candida albicans*, and *Trichophyton rubrum* compared to EOs of some high-mountain Himalayan *Nepeta* species rich in iridoid compounds [11]. In the present study, the antimicrobial activity of *N. subsessilis* and *N. govaniana* leaf methanol extracts were comparatively tested against 1 fungal and 10 bacterial pathogens (Table 3).

Although the extracts of both species possessed much lower antimicrobial activity compared to standard antimicrobial agents (ampicillin/caspofungin), the obtained results indicate that *N. govaniana* possesses stronger antimicrobial activity towards methicillin-resistant *S. aureus* (IBRS MRSA 011), *Listeria monocytogenes* ATCC 15313, and yeast *Candida auris* CDC B11903 compared to that of *N. subssesilis*. Such results could be ascribed to a higher content of nepetalactones in the *N. govaniana* leaf extract.

Moderate antimicrobial activity of the two species could possibly be ascribed to metabolic profiles characterized by low nepetalactone content, which are modulated, at least partially, by either the type of extraction procedure or the growth conditions. It is well known that growth conditions can shape the metabolic profiles of *Nepeta* species [18,134,135,136] In this study, *N. subsessilis* and *N. govaniana* individuals are cultivated under controlled growth conditions, and thus their metabolomes were independent of various geographical, seasonal, and environmental factors. It cannot be excluded that such growth conditions may reduce the accumulation of antimicrobial constituents (e.g., nepetalactones and phenolic compounds) in cultivated plants compared with naturally grown plants. On the other hand, the rationale for using methanol was its suitability to extract polar compounds (e.g., polyphenols and iridoid glycosides), but also semi-polar compounds such as nepetalactones, although with lower efficiency. In this way, we extracted compounds with strong antioxidant (polyphenolics) and antimicrobial activity (nepetalactone-type iridoids, iridoid glycosides, and polyphenolics), which would be probably more pronounced if less polar solvents were used. A number of studies reported potent antimicrobial activities of EOs [11,137,138,139,140,141] and methanol extracts of *Nepeta* species rich in nepetalactone-type iridoids [16,142,143].

Due to its richness in polyphenolic content and stronger antioxidant activity, *N. govaniana* may represent a promising source of antioxidative compounds. Moreover, its stronger antimicrobial activity, which may be associated with higher polyphenolic content, as well as with the higher abundance of nepetalactones, makes it a promising candidate for the development of antimicrobial agents. Although *N. subsessilis* exhibited lower antioxidant and antimicrobial activities compared to *N. govaniana*, this species has also demonstrated significant antimicrobial properties, indicating it may also serve as a valuable source of bioactive compounds.

## 3. Materials and Methods

### 3.1. Chemicals and Reagents

Acetonitrile (Fisher Scientific UK, Leicestershire, UK) and formic acid (Merck, Darmstadt, Germany) were of MS grade. Ultra-pure deionized water was generated using a Water Purification System (New Human Power I Integrate, Human Corporation, Seoul, Republic of Korea). Analytical standards of *trans,cis*-nepetalactone, 1,5,9-*epi*-deoxyloganic acid, and 5,9-dehydronepetalactone were isolated from natural sources as previously described by Aničić et al. (2021) [16]. Standards of *cis,trans*-nepetalactone and dihydronepetalactone were a generous gift from Entomol Products LLC (San Francisco, CA, USA). Analytical standards of loganin and aucubin were purchased from Sigma-Aldrich (Hamburg, Germany). Terpene Mix B (Merck KGaA, Darmstadt, Germany) and alkane standards (Supelco^®^, Bellefonte, PA, USA) were used for GC/MS identification of volatile compounds.

### 3.2. Plant Material

Seeds of *N. subsessilis* and *N. govaniana* were commercially purchased in 2024 from Jelitto Staudensamen GmbH (Schwarmstedt, Germany) and germinated in 2025 under greenhouse conditions, at the relative humidity of 60–70% and temperature of 25 ± 2 °C, in pots containing “BVB tray” soil (Kekkilä-BVB, Vantaa, Finland). One-month-old seedlings were transferred into pots containing “Floradur B cutting” soil (Floragard Product, Oldenburg, Germany) and grown for additional two months in the greenhouse at the Institute for Biological Research “Siniša Stanković”—National Institute of the Republic of Serbia, University of Belgrade (Serbia). All the experiments were performed in three biological replicates. Leaves from three-month-old *Nepeta* individuals were excised from flowering plants at midday and immediately soaked in liquid nitrogen (LN). Plant material was powdered using LN, weighted, and stored at −80 °C until used for the extraction of methanol-soluble metabolites, proteins, and DNA.

### 3.3. Extraction of Methanol-Soluble Metabolites

The extraction procedure included soaking of the samples in 96% methanol (w:v = 1:10), vortexing for 1 min, and ultrasound-assisted extraction for 1 h at 4 °C. Following centrifugation at 10,000× *g* for 10 min, supernatants were filtered through 0.22 µm filters (Agilent Technologies, Santa Clara, CA, USA) into a glass vials. Samples were stored at 4 °C until being analyzed. Leaf samples were made in triplicate for each *Nepeta* species.

### 3.4. Untargeted HPLC/Orbitrap MS Metabolomics

The identification of the compounds of interest in the extracts of the two *Nepeta* species was performed using the Thermo Scientific™ Vanquish™ Core HPLC system coupled to an Orbitrap Exploris 120 mass spectrometer (San Jose, CA, USA). Separation was achieved on a Hypersil Gold C18 column (San Jose, CA, USA) (50 × 2.1 mm, 1.9 μm) with a mobile phase that consisted of 0.1% formic acid in water (A) and acetonitrile +0.1% formic acid (B) at a flow rate of 0.3 mL min^−1^, using a 5 μL injection volume. The other LC/MS parameters were the same as previously published by Stojković et al. (2024) [144]. Semi-quantitative HPLC/Orbitrap MS data (peak areas) were subjected to hierarchical cluster analysis (HCA), using Spearman’s method of cluster agglomeration.

### 3.5. Untargeted GC/MS Metabolomics

Volatile terpenoids present in methanol leaf extracts of *N. subsessilis* and *N. govaniana* were profiled using an Agilent 8890 gas chromatograph (GC) coupled with a Mass Selective Detector (5977B GC/MSD, Agilent Technologies, Santa Clara, CA, USA) and connected to an automated sample extraction and enrichment platform (Centri^®^, Markes International Ltd., Bridgend, UK). The chromatographic separation conditions and MS parameters were previously described in detail by Banjanac et al. [8]. A 1 μL methanol extract was injected in a split mode (20:1), with a split flow of 24 mL min^−1^. Metabolites were chromatographically separated through an HP-5MS column (30 m × 0.25 mm, 0.25 μm film thickness) (Agilent Technologies, USA) using helium (99.999% purity, The Linde Group, Dublin, Ireland) as the carrier gas at a flow rate of 1.6 mL min^−1^. The temperature of the transfer line was set to 280 °C, while the detector temperature and the EI source temperatures were 270 °C and 280 °C, respectively. Mass spectra were acquired in the positive EI mode (+70 eV). The column temperature was linearly increased from 40 °C to 300 °C at the rate of 20 °C min^−1^ and held isothermally at 300 °C for 10 min. The analyses were performed in the SCAN mode, tracking compounds within the range of 45 to 500 amu. The constituents of the methanol extracts were identified using an Agilent Mass Hunter Workstation and the Unknown Analysis program by comparing the mass spectra and retention times of the compounds with those of the respective standards and with the NIST05 library, as previously described [8]. Kovats retention indices (RIs) were calculated according to the definition of Van Den Dool and Kratz (1963) [145], based on the elution characteristics of the alkane standards. To visualize the phytochemical differences in the content of volatile organic compounds (VOCs) between the two *Nepeta* species, semi-quantitative GC/MS data (peak areas) were subjected to HCA, using Pearson’s method of cluster agglomeration.

### 3.6. Isolation of Genomic DNA, Amplification and Sequencing of Plastid Loci

Total genomic DNA was isolated from fresh leaves of *N. govaniana* (Wall. ex Benth.) Benth. and *N. subsessilis* Maxim. using cetyltrimethylammonium bromide (CTAB) protocol as described by Doyle and Doyle (1990) [146], with minor modifications. Concentration and purity of the isolated DNA were determined spectrophotometrically (NanoPhotometer N60, IMPLEN, München, Germany) and fluorometrically (Qubit 3.0 fluorometer, Thermo Fisher Scientific, Waltham, MA, USA). In addition, gel electrophoresis was used to check DNA integrity.

### 3.7. PCR Amplification and Sequencing of Plastid Loci

PCR amplifications were performed using an Eppendorf Mastercycler nexus gradient thermal cycler (Eppendorf AG, Hamburg, Germany), with loci-specific settings presented in Table S4. The amplification mixture, in a final volume of 25 µL, contained 50 ng of template DNA, 12.5 µL Dream Taq Green PCR Master Mix (2×) (Thermo Fisher Scientific, Karlsruhe, Germany), and 0.2 µM both forward and reverse primers (Table S4).

The amplified PCR products were purified using off-the-shelf absorption, washing, and elution DNA buffers, and EconoSpin^®^ DNA spin columns (Epoch Life Science, Inc., Missouri City, TX, USA), as instructed by the manufacturer. Quality of the amplicons was checked by running the purified PCR products on ethidium bromide-stained 1% agarose gels. The gels were visualized in a UV transilluminator (ST4 3026-WL/26M, Vilber Lourmat, Collégien, France) and the length of amplicons was evaluated with reference to the 100 bp DNA Ladder Plus (Thermo Fisher Scientific, Karlsruhe, Germany).

Sequencing was performed on a SeqStudio™ Genetic Analyzer (Applied Biosystems, Thermo Fisher Scientific, Waltham, MA, USA). The obtained results were visualized and analyzed using Chromas (version 2.6.6, http://technelysium.com.au/wp/, accessed on 23 January 2026) and GeneMapper™ (Thermo Fisher Scientific, Waltham, MA, USA). Sequences were submitted to GenBank (accession numbers provided in Table S3).

### 3.8. Reconstruction of Phylogenetic Relations

Sequence alignment and phylogenetic analyses were performed using Molecular Evolutionary Genetics Analysis—MEGA, v.11.0.13. Alignment was performed using the MUSCLE algorithm with default settings. For phylogenetic analyses of the aligned sequences, the neighbor-joining method and the Tamura 3-parameter model (model with the lowest Bayesian information criterion value) were used. A bootstrap analysis with 1000 replicates was performed to evaluate the statistical support of branching.

The entire analysis included new sequences generated within this study along with the sequences of the *trn*L-F, *rbc*L, and *mat*K loci obtained from the NCBI database (for *N. cataria*, *N. ernesti-mayeri*, *N. grandiflora*, *N. laevigata*, *N. nervosa*, *N. nuda*, *N. parnassica*, *N. rtanjensis*, and *N. sibirica*) and publicly available plastid genomes of other *Nepeta* taxa (Table S3). The representatives of other genera belonging to the subclade Nepetoideae (*Salvia, Dracocephalum*, *Lavandula*, *Mentha*, and *Origanum*) were included into the analyses as outgroups.

### 3.9. Protein Extraction and Determination of Total Protein Content

Leaf soluble proteins were extracted using the procedure described by Dmitrović et al. (2015) [147] with slight modifications. About 100 mg of plant tissues were ground with liquid nitrogen and 1 mL of cold extraction buffer containing 100 mM Tris (pH 8.0), ethylenediaminetetraacetic acid (EDTA), 30% glycerol, 1.5% (*w*/*v*) polyvinylpyrrolidone (PVPP), 10 mM dithiothreitol (DTT), and 1 mM phenylmethylsulfonyl fluoride (PMSF) was added. Plant homogenate was centrifuged at 12,000× *g* for 15 min at 4 °C. The supernatants were collected, stored at −70 °C, and used for soluble protein determination and enzyme activity assays. Protein concentrations were measured using a Qubit^®^ 3.0 fluorometer (Thermo Fisher Scientific, Waltham, MA, USA).

### 3.10. SDS-PAGE and Immunoblotting

Protein samples for sodium dodecyl sulfate–polyacrylamide gel electrophoresis (SDS-PAGE) were mixed with an equal volume of the Laemmli buffer. Samples were heated at 95 °C for 3 min, rapidly cooled to 4 °C, and centrifuged at 10,000× *g* for 3 min. Protein separation was carried out at a room temperature using the Mini-Protein II system (Bio-Rad, Richmond, CA, USA) for 50 min at 200 V. Equal amounts of protein (15 μg) were loaded onto a 10% SDS–polyacrylamide separating gel and a 5% stacking gel. To estimate molecular weights, a prestained protein ladder ranging from 10 to 260 kDa (Spectra™ Multicolor Broad Range Protein Ladder, Thermo Fisher Scientific, Germany) was used. Following electrophoresis, the proteins were transferred onto PVDF membranes (Bio-Rad, USA) at 60 V for 1.5 h at 4 °C using a Mini Trans-Blot Module (Bio-Rad, USA). The immunoblot analysis was conducted using rabbit polyclonal antibodies anti-CAT (AS09501, Agrisera, Sweden), anti-PPO (AS10 1585, Agrisera, Sweden) or sheep polyclonal antibodies for POX (AS09548, Agrisera, Sweden), followed by a goat anti-rabbit IgG conjugated with horseradish peroxidase (A0545; Sigma-Aldrich, St. Louis, MO, USA). Protein bands were visualized using an enhanced chemiluminescence (ECL) detection system and quantified densitometrically using the ImageJ software (version 1.32j; W. Rasband, National Institutes of Health, USA). To confirm equal loading in immunoblots, membranes were incubated for 2 h in primary anti-ACT antibody (Anti rabbit Polyclonal lyophilized, AS13 2640; Agrisera Antibodies, Sweden) using the procedure described above. The obtained signal intensities for CAT, POX, and PPO were normalized to the actin values, and the obtained results were normalized to the highest value and presented as relative abundances.

### 3.11. Activities of Antioxidant Enzymes

The total activities of CAT, POX, were measured as described by Dmitrović et al. [147], while PPO activity was determined according to Živković et al. [148]. The rate of absorbance change was monitored using an Agilent 8453 spectrophotometer (Life Sciences, Santa Clara, CA, USA). The results are represented in triplicates, as specific activity units per milligram of total protein (U mg^−1^).

### 3.12. Antioxidant Capacity

The ferric ion-reducing antioxidant power (FRAP), DPPH and ABTS radical cation decolorization assays were determined spectrophotometrically [149]. For all three assays, methanol solutions of gallic acid (GA) were used for the calibration curve construction and the results are expressed as GA equivalents reducing activity (mg GAE) per 100 mg leaf FW. All analyses were performed in triplicate and absorbance measurements were recorded using a UV–Vis spectrophotometer (Agilent 8453, Agilent Technologies, Waldbronn, Germany).

### 3.13. Antimicrobial Assay

The antimicrobial assays were carried out using a modified microdilution method according to Smiljković et al. [29]. Bacterial strains were cultured overnight at 37 °C in tryptic soy broth, after which they were adjusted with sterile saline to a concentration of 1.0 × 10^5^ CFU mL^−1^. Samples dissolved in a 30% solution of ethanol were added to the tryptic soy broth (TSB) medium (100 μL) with bacterial inoculum (1.0 × 10^4^ CFU per well). After incubation, (24 h at 37 °C), iodonitrotetrazolium chloride (40 μL, 0.2 mg mL^−1^) was added to each well of the plate and further incubated for 60 min at 37 °C for the color development. The lowest concentrations that showed a distinct reduction in color intensity—light red in comparison to the intensive red in the control well (with no added test samples), were scored as minimal inhibitory concentrations (MICs). The minimal bactericidal concentrations (MBCs) were determined by a serial subcultivation of 2 μL into the wells already containing 100 μL of broth and further incubation for 24 h at 37 °C. The lowest concentration with no visible growth was recorded as the MBC, indicating 99.5% killing of the original inoculum.

Test microorganisms used involved Gram-positive bacteria, including methicillin-resistant Staphylococcus aureus (IBRS MRSA 011), Staphylococcus aureus (ATCC 11632), Bacillus cereus (food isolate), Listeria monocytogenes (NCTC 6890, ATCC 15313), and Staphylococcus lugdunensis (clinical isolate), Gram-negative bacteria, including Enterobacter cloacae (ATCC 35030), Escherichia coli O157:H7 (ATCC 700728 and ATCC 11775), and Escherichia coli (ATCC 35210), and the yeast Candida auris (CDC B11903) as a fungal test organism. Ampicillin was used as the positive control for the tested bacteria, while caspofungin served as the positive control for the tested yeast. The microorganisms used were obtained from the Mycological Laboratory, Department of Plant Physiology, Institute for Biological Research “Siniša Stanković”—National Institute of the Republic of Serbia, University of Belgrade.

### 3.14. Statistical Analyses

To compare the antioxidant activity between the samples, Student’s *t*-test was used. HCA plots based on qualitative metabolomics data were developed based on the method of cluster agglomeration and constructed in the Morpheus software [150]. Principal component analysis (PCA) was performed using the Past 4 software (version 4.14; Hammer and Harper, 2001).

## 4. Conclusions

This study provides a comprehensive comparative assessment of the phytochemical composition, biological activities, and phylogeny of *Nepeta govaniana* and *Nepeta subsessilis*. This study represents the first comprehensive characterization of iridoid aglycones and iridoid glycosides in *N. govaniana* and *N. subsessilis*, and provides a multidimensional phytochemical dataset by the parallel profiling of major metabolite classes using complementary analytics. Beyond expanding the known chemodiversity of the genus, it integrates metabolomic profiling with plastid DNA barcode-based phylogenetics and maps iridoid diversity onto the resulting evolutionary framework, providing new insights into the chemotaxonomic significance and evolutionary diversification of iridoid biosynthesis within *Nepeta*. These data revealed the phylogenetic relatedness of *N. govaniana* and *N. subsessiliss* with congeneric species, and placed them within the *Nepeta’s* chemotype A, whose members produce both iridoid aglycones and glycosylated iridoids.

*N. govaniana* exhibits significantly stronger enzymatic antioxidant capacity than *N. subsessilis*, with higher activities of CAT, PPO, and to a lesser extent POX. A higher antioxidant capacity of *N. govaniana* compared to *N. subsessilis* extract may be attributed to higher quantities of polyphenolics. Both methanolic extracts demonstrated moderate broad-spectrum antimicrobial activity against representative 10 bacterial and 1 fungal pathogen strains. Although the extracts of both species possessed much lower antimicrobial activity compared to the standard antimicrobial agents ampicillin and caspofungin, *N. govaniana* extract showed stronger antimicrobial activity towards methicillin-resistant *S. aureus* (IBRS MRSA 011), *Listeria monocytogenes* ATCC 15313, and yeast *Candida auris* CDC B11903 than *N. subssesilis*. The stronger antimicrobial potential of *N. govaniana* may be ascribed to a higher content of nepetalactones in the leaf extract. Overall, the practical significance of this study stems from the discovery of pronounced antimicrobial and antioxidant activities, particularly in *N. govaniana*, highlighting these species as promising sources of natural bioactive compounds for pharmaceutical, nutraceutical, and functional-product applications.

## Figures and Tables

**Table 1:** HPLC/Orbitrap MS data on metabolites identified in methanol extracts of *N. subsessilis* (*NS*) and *N. govaniana* (*NG*) leaves.

No	Compound Name	*t*_R_, min	Molecular Formula, [M–H]^−^/[M+H]+	Calculated Mass, *m*/*z*	Exact Mass, *m*/*z*	Δ mDa	MS^2^ Fragments, (% Base Peak)	*NS*	*NG*	Previosly Found in *Nepeta* or Lamiaceae
***Hydroxybenzoic acids***										
**1**	**Galloyl hexoside**	0.50	C_13_H_15_O_10_^−^	331.06707	331.06708	−0.01	125.02442(57), 168.00641(97), 169.01408(17), 313.05658(24), **331.06702**(100)	☑	☑	[15]
**2**	**Hydroxybenzoyl hexoside 1**	0.61	C_13_H_15_O_8_^−^	299.07724	299.07750	−0.26	93.03455(21), **137.02440**(100), 161.02420(4)	☑	☑	[16]
**3**	**Vanilloyl hexoside**	0.62	C_14_H_17_O_9_^−^	329.08781	329.08809	−0.28	108.02177(15), 123.04520(37), 152.01161(23), **167.03502**(100)	☑	☑	[17]
**4**	**Dihydroxybenzoyl hexoside 1**	0.65	C_13_H_15_O_9_^−^	315.07216	315.07242	−0.27	108.02166(40), 109.02952(57), 152.01152(88), **153.01933**(100), 315.0722(34)	☑	☑	[18]
**5**	**Syringoyl pentoside**	0.80	C_14_H_17_O_9_^−^	329.08781	329.08807	−0.26	153.05579(68), 182.02211(39), **197.04558**(100)	☑	☑	NA
**6**	**Hydroxybenzoic acid**	1.22	C_7_H_5_O_3_^−^	137.02442	137.02445	−0.03	93.03457(2), **137.02443**(100)	☑	☑	[19]
**7**	**Dihydroxybenzoyl hexoside 2**	4.36	C_13_H_15_O_9_^−^	315.07216	315.07229	−0.14	109.02969(11), **153.01932**(100)	☑	☒	[18]
**8**	**Dihydroxybenzoyl-glutaroyl hexoside**	4.63	C_18_H_21_O_12_^−^	429.10385	429.10374	0.11	108.02175(9), 109.02953(14), 152.01155(51), 153.01936(33), **315.07214**(100)	☑	☑	NA
**9**	**Hydroxybenzoyl hexoside 2**	4.64	C_13_H_15_O_8_^−^	299.07724	299.07725	−0.01	93.03461(38), **137.02441**(100)	☑	☑	[16]
**10**	**Dihydroxybenzoyl-syringoyl pentosyl-hexoside**	4.87	C_27_H_31_O_17_^−^	627.15667	627.15678	−0.11	108.02159(8), **152.01154**(100), 183.02998(45), 197.04556(31), 429.10355(54), 447.11490(20)	☒	☑	[20]
**11**	**Ethyl galloyl-galloyl hexoside**	5.10	C_22_H_23_O_14_^−^	511.10937	511.10986	−0.49	125.02444(34), **168.00644**(100), 169.01385(11), 197.04503(18), 331.06995(10), 493.09830(11)	☑	☑	NA
**12**	**Dihydroxybenzoyl-syringoyl hexoside**	5.28	C_22_H_23_O_13_^−^	495.11442	495.11429	0.12	109.02966(23), **152.01166**(100), 153.01942(42), 183.02982(24), 197.04539(28)	☒	☑	[21]
**13**	**Dihydroxybenzoyl-benzoyl hexoside**	6.20	C_20_H_19_O_10_^−^	419.09837	419.09820	0.17	108.02174(78), 109.02964(17), **152.01160**(100), 153.01938(40)	☒	☑	[21]
**14**	**Dihydroxybenzoyl-hydroxybenzoyl hexoside**	6.35	C_20_H_19_O_11_^−^	435.09329	435.09321	0.07	109.02946(8), **137.02449**(100), 152.01163(71), 153.01945(38), 297.06195(18), 315.07245(34)	☒	☑	[22]
***Hydroxycinnamic acids***										
**15**	**4-*O*-Feruloylquinic acid 1**	0.48	C_17_H_19_O_9_^−^	367.10346	367.10348	−0.02	93.03458(26), 111.04519(7), 134.03732(7), 173.04558(20), **191.05612**(100), 193.05051(23)	☑	☒	[23]
**16**	**Caffeoyltartaric acid 1**	0.49	C_13_H_11_O_9_^−^	311.04086	311.04108	−0.23	135.04529(32), **149.00923**(100), 179.03506(71)	☑	☑	[24]
**17**	**Salvianic acid A hexoside**	0.62	C_15_H_19_O_10_^−^	359.09837	359.09858	−0.21	123.04525(3), 135.04521(3), 179.03505(26), **197.04565**(100)	☑	☑	[25]
**18**	**4-*O*-Caffeoylquinic acid**	0.77	C_16_H_17_O_9_^−^	353.08781	353.08790	−0.09	135.0452(38), **173.04556**(100), 179.03500(76), 191.05614(68)	☒	☑	[26]
**19**	**5-*O*-Caffeoylquinic acid**	1.19	C_16_H_17_O_9_^−^	353.08781	353.08786	−0.05	173.04558(8), 179.03508(7), **191.05606**(100)	☒	☑	[16]
**20**	**Coumaroyltartaric acid 1**	1.38	C_13_H_11_O_8_^−^	295.04594	295.04604	−0.10	119.05032(35), **163.04016**(100)	☑	☒	NA
**21**	***p*** **-Coumaroy hexoside**	1.52	C_15_H_17_O_8_^−^	325.09289	325.09302	−0.13	119.05025(59), **163.04008**(100)	☑	☒	[27]
**22**	**Caffeic acid**	1.94	C_9_H_7_O_4_^−^	179.03498	179.03501	−0.03	**135.04520**(100), 179.03499(18)	☑	☑	[28]
**23**	**4-*O*-Feruloylquinic acid 2**	4.27	C_17_H_19_O_9_^−^	367.10346	367.10346	0.00	173.04559(16), **191.05614**(100), 193.05066(10)	☒	☑	[23]
**24**	**Feruloylglycolic acid**	4.59	C_12_H_11_O_6_^−^	251.05611	251.05612	0.00	134.03737(76), 149.06136(5), 178.02718(4), **193.05070**(100)	☒	☑	NA
**25**	**Lithospermic acid**	4.84	C_27_H_21_O_12_^−^	537.10385	537.10417	−0.32	135.04521(18), 161.02455(25), 179.03503(47), 197.04567(13), 269.08209(11), **295.06128**(100)	☒	☑	[29]
**26**	***p*** **-Coumaric acid**	4.86	C_9_H_7_O_3_^−^	163.04007	163.04012	−0.05	119.05025(16), **163.03986**(100)	☑	☑	[30]
**27**	**Yunnaneic acid D**	4.98	C_27_H_23_O_12_^−^	539.11950	539.12012	−0.62	135.04523(84), **161.02449**(100), 179.03506(69), 197.04562(53), 295.06085(20), 297.06924(32)	☒	☑	[31]
**28**	**Dicaffeoyltartaric acid**	5.04	C_22_H_17_O_12_^−^	473.07255	473.07247	0.08	135.04526(12), 149.00920(94), **179.03502**(100)	☒	☑	[32]
**29**	**Feruloyl tetroside**	5.11	C_14_H_15_O_7_^−^	295.08233	295.08239	−0.06	119.03475(24), 134.03722(44), 149.06120(12), 163.03996(18), 178.02757(8), **193.05072**(100)	☒	☑	NA
**30**	**Caffeoyltartaric acid 2**	5.14	C_13_H_11_O_9_^−^	311.04086	311.04093	−0.08	135.04529(23), **149.00920**(100), 179.03505(47)	☒	☑	[24]
**31**	**Feruloylmalic acid**	5.18	C_14_H_13_O_8_^−^	309.06159	309.06171	−0.11	115.00372(12), 134.03738(71), 149.06093(14), 178.02718(24), **193.05066**(100)	☑	☑	NA
**32**	**Salviaflaside**	5.35	C_24_H_25_O_13_^−^	521.13007	521.13021	−0.14	135.04518(91), **161.02446**(100), **179.03494**(100), 197.04556(39)	☑	☑	[33]
**33**	***p*** **-Coumaroylshikimic acid**	5.35	C_16_H_15_O_7_^−^	319.08233	319.08245	−0.12	111.04518(13), 119.05025(49), 137.02444(31), 145.02957(15), 155.03511(12), **163.04010**(100)	☒	☑	[34]
**34**	**Salviaflaside methyl ester**	5.36	C_25_H_27_O_13_^−^	535.14572	535.14577	−0.06	**135.04517**(100), 161.02417(5), 175.03983(10), 179.03513(9), 193.05061(12), 197.04544(14)	☑	☒	[33]
**35**	**Hydroxybenzoyl-caffeoyl hexoside**	5.38	C_22_H_21_O_12_^−^	477.10385	477.10444	−0.59	152.01157(43), 153.01962(22), **161.02446**(100), 179.03500(15), 315.07239(97), 323.07736(16)	☑	☑	NA
**36**	**Yunnaneic acid I**	5.45	C_27_H_25_O_12_^−^	541.13515	541.13544	−0.29	109.02950(36), **135.04518**(100), 179.03497(40), 193.05086(10), 197.04556(56), 299.09241(18)	☒	☑	[35]
**37**	**Dicaffeoylquinic acid**	5.48	C_25_H_23_O_12_^−^	515.11950	515.11970	−0.20	135.04520(12), 161.02457(4), **173.04555**(100), 179.03499(83), 191.05615(39), 353.08771(14)	☑	☑	[16]
**38**	**Salvianolic acid B**	5.48	C_36_H_29_O_16_^−^	717.14611	717.14621	−0.10	133.02946(15), 135.04514(9), 151.03999(29), **161.02435**(100), 179.03491(26), 197.04547(24)	☑	☑	[26]
**39**	**Caffeoyl-feruloyltartaric acid**	5.53	C_23_H_19_O_12_^−^	487.08820	487.09031	−2.11	134.0374(13), 135.04524(28), 149.00920(30), 161.02454(12), 179.03500(57), **193.05064**(100)	☒	☑	NA
**40**	**Feruloyl pentoside**	5.55	C_15_H_17_O_8_^−^	325.09289	325.09305	−0.16	134.03734(72), 149.04588(22), 149.06122(17), **193.05063**(100)	☑	☒	NA
**41**	**Caffeoyl hexoside**	5.56	C_15_H_17_O_9_^−^	341.08781	341.08781	−0.01	135.04523(26), **179.03499**(100)	☑	☒	[36]
**42**	**Feruloyltartaric acid**	5.56	C_14_H_13_O_9_^−^	325.05651	325.05666	−0.15	134.03735(37), 149.06085(14), 178.02687(4), **193.05061**(100)	☒	☑	[37]
**43**	**Coumaroyltartaric acid 2**	5.62	C_13_H_11_O_8_^−^	295.04594	295.04600	−0.05	119.05021(37), 149.00932(30), **163.04007**(100), 193.05164(6)	☒	☑	NA
**44**	**Caffeoyl-coumaroyltartaric acid**	5.62	C_22_H_17_O_11_^−^	457.07764	457.07766	−0.02	151.04008(35), 161.02446(51), **163.04010**(100), 179.03505(33), 197.04512(19), 219.02991(19)	☒	☑	NA
**45**	**Ferulic acid**	5.72	C_10_H_9_O_4_^−^	193.05063	193.05064	−0.01	109.02934(2), 133.02943(4), 134.03738(35), 161.02446(20), 178.02721(5), **193.05061**(100)	☑	☑	[28]
**46**	**Rosmarinic acid**	5.72	C_18_H_15_O_8_^−^	359.07724	359.07727	−0.03	135.04515(7), **161.02440**(100), 179.03497(20), 197.04555(34)	☑	☑	[28]
**47**	**Salvianic acid A**	5.72	C_9_H_9_O_5_^−^	197.04555	197.04555	0.00	123.04516(59), **135.04517**(100), 179.03494(44), 197.04553(7)	☒	☑	[38]
**48**	**Sagerinic acid**	5.73	C_36_H_31_O_16_^−^	719.16176	719.16144	0.32	135.04518(4), **161.02444**(100), 179.03503(10), 197.04556(39)	☒	☑	[39]
**49**	**Caffeoyl-coumaroyl hexoside**	5.75	C_24_H_23_O_11_^−^	487.12459	487.12461	−0.02	119.05025(16), 135.04514(14), **161.02438**(100), 163.04001(40), 179.03490(52), 323.07712(31)	☑	☑	NA
**50**	**Skimmin**	5.86	C_15_H_15_O_8_^−^	323.07724	323.07732	−0.07	135.04506(4), 143.03493(96), 161.02704(3), **161.04549**(100), 179.03635(4)	☒	☑	[23]
**51**	**Coumaroyl-feruloyltartaric acid**	5.94	C_23_H_19_O_11_^−^	471.09329	471.09310	0.19	119.05024(71), 145.02950(23), **163.04005**(100), 175.04071(10), 193.05040(28), 203.03497(88)	☒	☑	NA
**52**	**Feruloyl-caffeoylquinic acid**	6.06	C_26_H_25_O_12_^−^	529.13515	529.13527	−0.12	135.04492(7), 161.02486(6), **173.04552**(100), 179.03476(28), 191.05649(24), 193.05072(18)	☒	☑	[40]
**53**	**Clinopodic acid A**	6.10	C_18_H_15_O_7_^−^	343.08233	343.08234	−0.01	135.0452(59), **145.02950**(100), 179.03497(29), 197.04559(45)	☑	☑	[16]
**54**	**Schizotenuin F**	6.14	C_28_H_23_O_12_^−^	551.11950	551.11942	0.08	133.02957(25), 135.04518(59), **161.02446**(100), 179.03503(23), 267.06625(31), 311.05612(57)	☑	☒	[41]
**55**	**Feruloyl-benzoyl hexuronide**	6.15	C_23_H_21_O_11_^−^	473.10894	473.10917	−0.23	134.03737(7), 135.03015(90), 161.02454(12), 175.03995(5), 179.03503(30), **193.05063**(100)	☑	☒	NA
**56**	**Sinapoyl-rosmarinoyl hexoside**	6.19	C_35_H_35_O_17_^−^	727.18797	727.18849	−0.51	135.04523(31), **161.02448**(100), 179.03503(48), 197.04561(29), 367.10297(10)	☑	☒	[42]
**57**	**Methyl rosmarinate**	6.56	C_19_H_17_O_8_^−^	373.09289	373.09301	−0.12	**135.04521**(100), 160.01662(10), 175.04008(51), 179.03500(25), 197.04562(37)	☑	☑	[16]
**58**	**Nepetoidin B or A**	6.68	C_17_H_13_O_6_^−^	313.07176	313.07181	−0.05	**161.02441**(100)	☑	☑	[16]
**59**	**Salvianolic acid C**	6.93	C_26_H_19_O_10_^−^	491.09837	491.09845	−0.08	135.04521(11), 161.02449(21), **179.03494**(100), 267.06616(11)	☑	☑	[43]
**60**	**Orthosiphoic acid A**	7.11	C_27_H_21_O_11_^−^	521.10894	521.10932	−0.38	**135.04514**(100), 163.06151(12), 179.03499(13), 309.04028(15), 327.05063(16), 341.06683(73)	☒	☑	[44]
**61**	**Methyl salvianolate C**	7.79	C_27_H_21_O_10_^−^	505.11402	505.11437	−0.35	149.02467(4), 161.02446(24), 163.06151(9), **193.05070**(100)	☑	☑	[45]
***Phenylethanoids***										
**62**	**Teupolioside**	5.08	C_35_H_45_O_20_^−^	785.25097	785.25110	−0.13	**161.02444**(100), 179.03481(6), 461.16608(31), 623.19788(18)	☑	☒	[46]
**63**	**Dehydroacteoside**	5.11	C_29_H_33_O_15_^−^	621.18249	621.18274	−0.24	113.02449(10), 135.04518(22), 151.04010(14), 161.02444(85), **179.03496**(100)	☑	☒	[47]
**64**	**Verbascoside**	5.30	C_29_H_35_O_15_^−^	623.19814	623.19837	−0.23	135.04524(9), **161.02440**(100), 179.03487(3), 315.10818(3), 461.16608(12)	☒	☑	[48]
**65**	**3‴-*O*-Methylcrenatoside**	5.58	C_30_H_35_O_15_^−^	635.19814	635.19823	−0.09	113.02431(14), 134.03731(24), 151.04022(20), 161.02432(21), 175.03998(43), **193.05052**(100)	☑	☒	[49]
**66**	**Diacetyl-verbascoside**	5.73	C_33_H_39_O_17_^−^	707.21927	707.21961	−0.34	**161.02440**(100), 179.03491(11), 197.04550(43)	☒	☑	[50]
**67**	**Kankanoside G**	5.88	C_29_H_35_O_14_^−^	607.20323	607.20270	0.53	113.02453(9), 145.0298(5), **161.02446**(100), 179.03476(4)	☒	☑	[51]
**68**	**Cuneataside D**	6.18	C_28_H_33_O_13_^−^	577.19267	577.19306	−0.39	101.02439(4), **161.02448**(100), 269.10239(4)	☑	☒	NA
**69**	**Premnethanoside A or B**	6.34	C_41_H_53_O_22_^−^	897.30340	897.30352	−0.12	134.03735(7), **175.04004**(100), 193.0506(29), 475.18134(6), 505.17139(9), 651.22937(8)	☑	☒	[52]
***Iridoid glycosides***										
**70**	**1-*O*-Hexosyl-*epi*-deoxyloganic acid**	4.49	C_22_H_33_O_14_^−^	521.18758	521.18759	−0.01	135.08162(53), **153.09215**(100), 161.04561(6), 197.08200(53), 341.12369(8), 359.13474(5)	☑	☑	[16]
**71**	**Adenosmoside**	4.51	C_16_H_27_O_9_^−^	363.16606	363.16543	0.62	**59.01385**(100), 89.02442(82), 101.02446(49), 113.02458(31), 119.03500(40), 183.10281(12)	☑	☑	[53]
**72**	**Nepetanudoside**	4.51	C_17_H_25_O_10_^−^	389.14532	389.14538	−0.06	**101.02443**(100), 183.06645(4), 227.09236(13)	☒	☑	[54]
**73**	**6α-Hydroxyadoxoside**	4.81	C_17_H_25_O_11_^−^	405.14024	405.14026	−0.03	**61.98836**(100), 109.06563(1), 135.08168(2), 153.09206(5), 197.08211(4)	☑	☑	[55]
**74**	**1-*O*-Pentosyl-*epi*-deoxyloganic acid**	5.00	C_21_H_31_O_13_^−^	491.17702	491.17719	−0.18	101.02440(8), 109.06594(13), 135.08162(59), **153.09212**(100), 197.08197(48)	☑	☒	[18]
**75**	**1,5,9-*Epi*-deoxyloganic acid**	5.06	C_16_H_23_O_9_^−^	359.13476	359.13481	−0.05	101.02454(4), 119.03487(4), 135.08153(94), **153.09209**(100), 197.08188(32)	☑	☑	[56]
**76**	**5-Deoxylamiol**	5.29	C_16_H_25_O_9_^−^	361.15041	361.15036	0.04	137.09740(13), **155.10789**(100), 181.08723(5), 199.09775(36)	☒	☑	[57]
**77**	**Nepetaracemoside A**	5.43	C_16_H_23_O_8_^−^	343.13984	343.13990	−0.06	109.06596(14), 135.08151(86), **153.09206**(100), 197.08185(33)	☑	☒	[58]
**78**	**Nepetaside**	5.53	C_16_H_25_O_7_^−^	345.15549	345.15549	0.00	**59.01384**(100), 101.02441(47), 113.02444(29), 119.03500(35), 183.10266(34), 345.15512(17)	☑	☑	[59]
**79**	**Nepetariaside**	5.79	C_16_H_27_O_8_^−^	347.17114	347.17115	−0.01	**59.01387**(100), 101.02446(50), 119.03504(40), 167.10783(15), 185.11850(5), 347.17105(38)	☑	☑	[60]
***Iridoid aglycones***										
**80**	**Deoxyloganetic acid**	4.63	C_10_H_15_O_4_^+^	199.09649	199.09516	1.32	**135.07944**(100), 137.09474(24), 151.07417(17), 153.08995(48), 163.07411(86), 181.08456(51)	☑	☒	[61]
**81**	**Nepetaracemoside B aglycone**	5.15	C_10_H_13_O_3_^+^	181.08592	181.08412	1.80	93.06895(18), 95.04823(10), 135.07918(32), 145.0271(12), **163.07368**(100), 181.08417(17)	☑	☒	[58]
**82**	**Nepetalactol**	5.40	C_10_H_17_O_2_^+^	169.12231	169.12110	1.20	81.06924(16), 93.06915(41), 95.08480(26), 123.11598(16), 133.10023(15), **151.11066**(100)	☑	☑	[8]
**83**	**Dihydronepetalactone**	5.57	C_10_H_17_O_2_^+^	169.12231	169.12118	1.13	81.06921(33), 95.08475(10), **123.11583**(100)	☑	☑	[62]
**84**	**Deoxygeniposide aglycone**	6.07	C_11_H_15_O_4_^+^	211.09649	211.09504	1.45	105.06906(30), 133.06372(47), **151.07411**(100), 165.08971(35), 179.06868(28), 193.08452(13)	☑	☑	[54]
**85**	**Dihydronepetalactone**	6.21	C_10_H_17_O_2_^+^	169.12231	169.12106	1.24	81.06922(23), 109.10023(4), **123.11583**(100)	☑	☒	[62]
**86**	**Nepetalic acid**	6.22	C_10_H_15_O_3_^−^	183.10267	183.10270	−0.03	139.11287(23), 155.10771(3), 165.09222(10), **183.10271**(100)	☑	☑	[63]
**87**	**Dehydronepetalactone**	6.84	C_10_H_13_O_2_^+^	165.09101	165.08986	1.14	**165.08980**(100)	☑	☑	[38]
**88**	***cis,trans*** **-Nepetalactone**	7.44	C_10_H_15_O_2_^+^	167.10666	167.10532	1.34	81.06924(12), 93.06915(32), **121.10019**(100), 123.07942(8), 125.09509(21), 139.11067(34)	☑	☑	[38]
**89**	***trans,cis*** **-Nepetalactone**	7.93	C_10_H_15_O_2_^+^	167.10666	167.10539	1.27	**81.06921**(100), 93.06909(11), 121.10023(32), 123.11585(27), 125.05864(26), 139.11082(10)	☑	☑	[64]
***Flavonid glycosides***										
**90**	**Apigenin 6-C-pentoside-7-O-hexoside**	4.66	C_26_H_27_O_14_^−^	563.14063	563.14112	−0.50	257.04831(32), 357.09982(19), 369.06369(16), 399.07498(29), **401.09070**(100), 443.09943(16)	☑	☒	[65]
**91**	**Luteolin 7-*O*-(2″-hexuronyl)-hexuronide**	4.47	C_27_H_25_O_18_^−^	637.10464	637.10476	−0.13	113.02442(28), 175.02478(7), 193.03534(17), **285.04041**(100), 351.05643(21)	☑	☒	[56]
**92**	**Apigenin 7-*O*-(2″-hexuronyl)-hexuronide**	4.89	C_27_H_25_O_17_^−^	621.10972	621.10992	−0.20	**113.02444**(100), 131.03520(12), 175.0249(23), 193.03548(44), 269.04538(52), 351.05588(34)	☑	☒	[66]
**93**	**Quercetin 3-*O*-(6″-rhamnosyl)-hexoside**	5.03	C_27_H_29_O_16_^−^	609.14611	609.14662	−0.51	**300.02777**(100), 301.03549(43)	☑	☑	[19]
**94**	**Luteolin 7-*O*-(6″-rhamnosyl)-hexoside**	5.15	C_27_H_29_O_15_^−^	593.15119	593.15166	−0.47	284.03256(3), **285.04047**(100)	☒	☑	[67]
**95**	**Quercetin 3-*O*-hexoside**	5.15	C_21_H_19_O_12_^−^	463.08820	463.08775	0.45	151.00389(3), 178.99890(3), **300.02762**(100), 301.03537(33)	☒	☑	[68]
**96**	**Quercetin 3-*O*-[6″-(3-hydroxy-3-methylglutaroyl)]-hexoside**	5.22	C_27_H_27_O_16_^−^	607.13046	607.13068	−0.22	151.00443(3), **300.02744**(100), 301.03525(52), 463.08771(18), 505.09879(9)	☑	☑	[69]
**97**	**Kaempferol 3-O-hexoside**	5.22	C_21_H_19_O_11_^−^	447.09329	447.09352	−0.23	255.02989(10), **284.03259**(100), 285.04030(27)	☑	☑	[70]
**98**	**Luteolin 7-*O*-hexuronide**	5.23	C_21_H_17_O_12_^−^	461.07255	461.07263	−0.08	113.02459(3), **285.04047**(100)	☑	☑	[70]
**99**	**Apigenin 7-*O*-hexoside**	5.52	C_21_H_19_O_10_^−^	431.09837	431.09877	−0.40	**268.03775**(100), 269.0455(78), 311.05576(3), 431.09821(88)	☑	☒	[24]
**100**	**Chrysoeriol 7-*O*-(6″-rhamnosyl)-hexoside**	5.59	C_28_H_31_O_15_^−^	607.16684	607.16729	−0.45	284.03265(21), **299.05609**(100)	☑	☒	[71]
**101**	**Apigenin 7-*O*-hexuronide**	5.62	C_21_H_17_O_11_^−^	445.07764	445.07773	−0.10	113.02446(15), **269.04559**(100)	☑	☑	[67]
**102**	**Kaempferol 3-*O*-[6″-(3-hydroxy-3-methylglutaroyl)]-hexoside**	5.67	C_27_H_27_O_15_^−^	591.13554	591.13580	−0.26	284.03253(76), **285.04034**(100), 447.09232(13), 489.10498(13), 529.13306(4), 547.10895(3)	☒	☑	[69]
**103**	**Luteolin 7-*O*-(6″-malonyl)-hexoside**	5.67	C_24_H_21_O_14_^−^	533.09368	533.09435	−0.67	255.03046(4), **284.03268**(100), 285.04056(86)	☒	☑	[72]
**104**	**Hesperetin 7-*O*-hexuronide**	5.87	C_22_H_21_O_12_^−^	477.10385	477.10430	−0.45	113.02457(37), 153.01973(14), 286.04593(12), **301.07153**(100)	☒	☑	NA
**105**	**Thymusin 6-*O*-hexoside**	6.20	C_23_H_23_O_12_^−^	491.11950	491.11971	−0.21	314.04303(38), **329.06650**(100)	☑	☑	[73]
**106**	**Luteolin 7-*O*-(4″-caffeoyl)-hexuronide**	6.24	C_30_H_23_O_15_^−^	623.10424	623.10449	−0.25	**161.02449**(100), 175.02466(5), 179.03520(6), 285.04062(55), 337.05609(5), 443.06213(14)	☑	☑	[74]
**107**	**Acacetin 7-*O*-(tri-pentosyl)-hexoside**	6.25	C_37_H_45_O_22_^−^	841.24080	841.24106	−0.27	268.03735(4), **283.06107**(100)	☑	☑	[75]
**108**	**Acacetin 7-*O*-(6″-rhamnosyl)-hexoside**	6.29	C_28_H_31_O_14_^−^	591.17193	591.17207	−0.14	268.03757(11), **283.06097**(100)	☒	☑	[24]
**109**	**Thymusin 6-*O*-(6″-acetyl)-hexoside**	6.47	C_25_H_25_O_13_^−^	533.13007	533.13031	−0.24	299.01999(9), 314.04288(40), **329.06656**(100)	☒	☑	NA
**110**	**Apigenin 7-*O*-(4″-caffeoyl)-hexuronide**	6.52	C_30_H_23_O_14_^−^	607.10933	607.10965	−0.32	145.02986(15), **161.02446**(100), 269.04587(12), 427.06110(14), 443.06180(19)	☑	☒	NA
**111**	**Chrysoeriol 7-*O*-hexoside**	6.54	C_22_H_21_O_11_^−^	461.10894	461.10930	−0.37	125.09715(4), 187.09798(5), 283.02484(24), 284.03171(12), **298.04837**(100), 299.05612(79)	☒	☑	[73]
**112**	**Luteolin 7-*O*-(4″-feruloyl)-hexuronide**	6.57	C_31_H_25_O_15_^−^	637.11989	637.12017	−0.28	161.02467(8), 175.04047(15), 193.05099(4), 284.03214(9), **285.04044**(100), 443.06201(41)	☒	☑	NA
**113**	**Chrysoeriol 7-*O*-[2″-(5′″-acetyl)-pentoyl]-pentoside**	6.63	C_28_H_29_O_15_^−^	605.15119	605.15161	−0.41	283.02515(7), 284.03326(4), 298.04813(78), **299.05588**(100)	☒	☑	[76]
***Flavonoid aglycones***										
**114**	**Baicalein**	5.62	C_15_H_9_O_5_^−^	269.04555	269.04558	−0.03	149.02467(2), **269.04550**(100)	☑	☒	NA
**115**	**Thymusin**	6.09	C_17_H_13_O_7_^−^	329.06668	329.06700	−0.32	271.02002(9), **299.01990**(100), 314.04318(69)	☑	☒	[77]
**116**	**Luteolin**	6.26	C_15_H_9_O_6_^−^	285.04046	285.04056	−0.10	209.02707(8), 211.04271(6), 239.03763(15), **241.05307**(100)	☑	☑	[67]
**117**	**Naringenin**	6.72	C_15_H_11_O_5_^−^	271.06120	271.06130	−0.11	107.01388(12), 119.05032(41), **151.00374**(100), 165.01961(4), 177.01930(11), 271.06122(54)	☑	☑	[38]
**118**	**Apigenin**	6.72	C_15_H_9_O_5_^−^	269.04555	269.04575	−0.20	149.02495(3), 151.00371(3), 225.05663(2), **269.04550**(100)	☑	☑	[38]
**119**	**Isothymusin**	6.91	C_17_H_13_O_7_^−^	329.06668	329.06684	−0.16	**299.01971**(100), 314.04318(77)	☑	☑	[38]
**120**	**Cirsimaritin**	7.40	C_17_H_13_O_6_^−^	313.07176	313.07187	−0.11	269.04578(5), **283.02478**(100), 297.04053(16), 298.04825(87), 313.07190(27)	☑	☑	[28]
**121**	**Chrysoeriol**	7.40	C_16_H_11_O_6_^−^	299.05611	299.05621	−0.10	284.03253(69), **299.05600**(100)	☑	☑	[77]
**122**	**Pedunculin**	7.74	C_18_H_15_O_7_^−^	343.08233	343.08234	−0.01	298.01196(6), **313.03534**(100), 328.05899(36)	☑	☑	[77]
**123**	**Acacetin**	7.91	C_16_H_11_O_5_^−^	283.06120	283.06130	−0.10	268.03775(90), **283.06122**(100)	☑	☑	[77]
***Fatty acids***										
**124**	**Hydroxy-octanedicarboxylic acid**	5.25	C_10_H_17_O_5_^−^	217.10815	217.10817	−0.02	137.09718(5), 155.10780(62), 171.10272(65), 199.09778(9), **217.10815**(100)	☑	☑	NA
**125**	**Hydroxy-undecanedioic acid**	5.62	C_11_H_19_O_5_^−^	231.12380	231.12399	−0.19	151.11281(3), 169.12344(31), **171.10275**(100), 213.1132(5), 231.12399(11)	☑	☑	NA
**126**	**Hydroxy-decenoic acid**	6.21	C_10_H_17_O_3_^−^	185.11832	185.11838	−0.07	57.03465(2), 139.11287(44), 141.12857(3), 167.10789(8), 185.11829(100)	☑	☑	NA
**127**	**Hydroxydodecanedioic acid**	6.48	C_12_H_21_O_5_^−^	245.13945	245.13949	−0.04	165.12843(8), 183.13922(58), 199.13406(59), 227.12828(7), **245.13960**(100)	☑	☑	NA
**128**	**9-Oxononanoic acid**	6.78	C_9_H_15_O_3_^−^	171.10267	171.10271	−0.04	125.09737(9), **127.11298**(100), 153.09233(13), 171.10280(60)	☑	☑	[78]
**129**	**Hydroxy-nonanoic acid**	7.43	C_9_H_17_O_3_^−^	173.11832	173.11832	0.00	111.08152(5), **127.11287**(100), 155.10779(3), 173.11829(72)	☑	☑	NA
**130**	**Hydroxy-dodecanoic acid**	9.15	C_12_H_23_O_3_^−^	215.16527	215.16527	0.00	**169.15999**(100), 197.15489(3), 215.16550(64)	☑	☑	NA
***Other compounds***										
**131**	**Methyl hexuronic acid**	0.50	C_7_H_11_O_7_^−^	207.05103	207.05119	−0.16	**135.04523**(100), 163.04025(8), 177.05574(5), 192.04288(40), 207.06647(6)	☑	☑	NA
**132**	**Shikimic acid**	0.50	C_7_H_9_O_5_^−^	173.04555	173.04560	−0.05	93.03461(71), 99.00874(13), 111.0452(28), 137.02444(12), 155.03523(7), **173.04558**(100)	☑	☑	NA
**133**	**Quinic acid**	0.52	C_6_H_7_O_7_^−^	191.01973	191.01977	−0.04	85.02953(10), 111.04525(2), 127.04017(3), 173.04538(2), **191.05615**(100)	☑	☑	[28]
**134**	**Hydroxyglutaric acid**	0.60	C_5_H_7_O_5_^−^	147.02990	147.02991	−0.01	85.02954(56), **87.00878**(100), 101.02448(6), 103.04012(11), 129.01945(21), 147.02998(65)	☑	☑	NA
**135**	**Glutaric acid**	0.61	C_5_H_7_O_4_^−^	131.03498	131.03508	−0.10	**87.04515**(100), 113.02454(7), 131.03506(34)	☑	☑	NA
**136**	**Ascorbic acid**	0.66	C_6_H_7_O_6_^−^	175.02481	175.02488	−0.07	**87.00880**(100), 113.02454(10), 147.03008(8)	☑	☑	[28]
**137**	**Tuberonic acid hexoside**	3.64	C_18_H_27_O_9_^−^	387.16606	387.16585	0.21	**59.01382**(100), 89.02435(31), 101.02439(19), 207.10257(27), 387.16553(37)	☑	☑	[26]
**138**	**8-Oxogeranial**	5.32	C_10_H_15_O_2_^+^	167.10666	167.10543	1.22	81.06922(20), **93.06921**(100), 111.07963(25), 121.1003(95), 139.11095(10)	☑	☒	[79]
**139**	**Elshrugulosain**	5.34	C_26_H_29_O_12_^−^	533.16645	533.16638	0.07	294.08871(30), 309.07516(35), **338.08051**(100), 341.10196(87), 353.10260(85)	☑	☑	[80]
**140**	**Citrusin D**	5.75	C_16_H_21_O_8_^−^	341.12419	341.12421	−0.02	164.04787(46), **179.07141**(100)	☑	☒	[81]
**141**	**Eugenol hexoside (Citrusin C)**	5.95	C_16_H_21_O_7_^−^	325.12929	325.12939	−0.10	71.01392(46), 85.02972(18), 101.02445(29), 113.02476(27), 148.05276(21), **163.07664**(100)	☑	☑	[82]
**142**	**2-Carboxy-α,3-dimethyl-cyclopentaneacetic acid**	6.16	C_10_H_15_O_4_^−^	199.09758	199.09760	−0.02	137.09734(6), **155.10786**(100), 199.09892(7)	☑	☑	[83]
**143**	**2-Carboxy-α,3-dimethyl-cyclopentaneacetic acid methyl ester**	6.21	C_11_H_17_O_4_^−^	213.11323	213.11331	−0.08	123.08149(17), 125.09704(4), **139.11287**(100), 151.11316(12), 169.12344(12), 213.11328(93)	☑	☑	[84]
**144**	**Argolic acid A**	6.35	C_10_H_17_O_4_^−^	201.11323	201.11336	−0.13	**139.11290**(100) 183.10271(79), 201.11330(57)	☑	☑	[85]
**145**	**2-Carboxy-3-methyl-cyclopentaneacetic acid**	6.38	C_9_H_13_O_4_^−^	185.08193	185.08198	−0.05	139.11284(4), **141.09219**(100), 185.08279(4)	☑	☑	[86]

NA—not available; ☒—undetected compound; ☑—detected compound.

**Table 2:** GC/MS profiling of VOCs in methanol extracts of *N. subsessilis* (*NS*) and *N. govaniana* (*NG*) leaves.

No.	tR (min)	Compound Assignment	RI	Chemical Formula	*NS*	*NG*
Monoterpene hydrocarbons						
**G1**	6.39	*α*-Pinene ^a^	935	C_10_H_16_	+	+
**G2**	6.82	Sabinene	978	C_10_H_16_	+	+
**G3**	6.87	*β*-Pinene ^a^	983	C_10_H_16_	+	+
**G4**	6.97	*β*-Myrcene	992	C_10_H_16_	/	+
**G5**	7.33	o-Cymene	1029	C_10_H_14_	+	/
**G6**	7.37	D-Limonene ^a^	1033	C_10_H_16_	+	+
Oxygenated monoterpenes						
**G7**	7.41	1,8-Cineole ^a^	1037	C_10_H_18_O	+	+
**G8**	8.6	*δ*-Terpineol	1056	C_10_H_18_O	+	+
**G9**	9.17	Pulegone	1239	C_10_H_16_O	+	+
**G10**	10.04	*trans*,*trans*-Nepetalactone	1326	C_10_H_14_O_2_	+	+
**G11**	10.09	*Unknow nepetalactone isomer 1*	1331	C_10_H_14_O_2_	+	/
**G12**	10.12	*cis*,*trans*-Nepetalactone ^a^	1334	C_10_H_14_O_2_	+	+
**G13**	10.30	*trans*,*cis*-Nepetalactone ^a^	1409	C_10_H_14_O_2_	+	/
**G14**	10.33	*cis,cis*-Nepetalactone ^a^	1465	C_10_H_14_O_2_	+	+
**G15**	11.09	5,9-Dehydronepetalactone ^a^	1482	C_10_H_12_O_2_	/	+
**G16**	11.93	Dihydronepetalactone	1647	C_10_H_16_O_2_	/	+
Sesquiterpene hydrocarbons						
**G17**	10.51	*β*-Caryophyllene ^a^	1430	C_15_H_24_	+	+
**G18**	10.73	Humulene	1452	C_15_H_24_	/	+
**G19**	10.9	Germacrene D ^a^	1469	C_15_H_24_	+	+
**G20**	10.94	*γ*-Himachalene	1473	C_15_H_24_	/	+
**G21**	10.99	Bicylogermacrene	1478	C_15_H_24_	/	+
Oxygenated sesquiterpenes						
**G22**	11.78	Humulene epoxide I	1632	C_15_H_24_O	/	+
Diterpene hydrocarbons						
**G23**	12.81	Neophytadiene 1	1822	C_20_H_38_	+	+
**G24**	12.85	2-Phytene	1826	C_20_H_38_	+	+
**G25**	12.94	Neophytadiene 2	1835	C_20_H_38_	+	+
**G26**	13.04	Neophytadiene 2	1845	C_20_H_38_	+	+
Oxygenated diterpenes						
**G27**	14.18	Phytol	2057	C_20_H_40_O	+	+

Abbreviations: RI, Kovats retention index; ^a^, confirmed by authentic standard; +, presence confirmed; /, presence not confirmed.

**Table 3:** Antimicrobial activity of *N. subsessilis* (*NS*) and *N. govaniana* (*NG*) methanol extracts, as revealed by microdilution technique. Values for minimal inhibitory (MIC) and lethal concentration (MBC-bactericidal and MFC-fungicidal concentration) are presented as mg of extract per mL (mg mL^−1^).

Microorganism		*NS*	*NG*	Ampicillin/ Caspofungin
IBRS MRSA 011	MIC	1.0	0.5	0.00160
	MBC	2.0	1.0	0.00320
*Staphylococcus aureus* ATCC 11632	MIC	0.5	0.5	0.00630
	MBC	1.0	1.0	0.01300
*Bacillus cereus* (food isolate)	MIC	0.5	0.5	0.00013
	MBC	1.0	1.0	0.00027
*Listeria monocytogenes* NCTC 7973	MIC	0.5	0.5	0.00014
	MBC	1.0	1.0	0.00028
*Listeria monocytogenes* ATCC 15313	MIC	1.0	1.0	0.00028
	MBC	2.0	1.0	0.00056
*Enterococcus cloacae* ATCC 35030	MIC	0.5	0.5	0.00022
	MBC	0.5	1.0	0.00043
*Escherichia coli* O157:H7 ATCC 700728	MIC	1.0	1.0	0.00015
	MBC	2.0	2.0	0.00029
*Escherichia coli* ATCC 35210	MIC	1.0	1.0	0.00015
	MBC	2.0	2.0	0.00029
*Escherichia coli* O157:H7 ATCC 11775	MIC	2.0	1.0	0.00015
	MBC	2.0	2.0	0.00029
*Staphylococcus lugdunensis* clinical isolate	MIC	1.0	1.0	0.00028
	MBC	2.0	2.0	0.00056
*Candida auris* CDC B11903	MIC	1.0	0.5	0.02500
	MFC	2.0	1.0	0.05000

## Data Availability

The original contributions presented in this study are included in the article/Supplementary Materials. Further inquiries can be directed to the corresponding authors.

## Supplementary Materials

The following supporting information can be downloaded at: https://www.mdpi.com/article/10.3390/plants15121804/s1, Figure S1: Heatmap of the scaled data of untargeted LC-MS metabolite analysis, with the samples (both columns and rows) arranged according to the HCA (Pearson method of cluster agglomeration). Intensity of green color indicate the abundance of the compounds in samples; Figure S2: Relative abundances of antioxidative proteins. The obtained signal intensities for CAT, POX and PPO were normalized to the actin values, and the obtained results were normalized to the highest value and presented as relative abundances; Table S1: Peak areas of the HPLC/Orbitrap MS data corresponding to metabolites identified in methanol extracts of *N. subsessilis* (*NS*) and *N. govaniana* (*NG*) leaves; Table S2: Peak areas of the metabolites identified in methanol extracts of *N. subsessilis* and *N. govaniana* leaves, as revealed by GC/MS analysis; Table S3: GenBank accession numbers for DNA sequences used in this study to reconstruct phylogenetic relationships, as presented in Figure 3; Table S4: Primer sequences and PCR conditions used for amplification of plastid loci.
