# Supplementary material for: Towards Understanding the Bioactive Potential of Two Intriguing Nepeta Species: Metabolomic and Phylogenetic Perspectives
Source: Plants (Basel). 2026 Jun 11;15(12):1804. doi: 10.3390/plants15121804 (PMC13306286; doi:10.3390/plants15121804)
Supplement: Supplementary file 1 [file plants-15-01804-s001.zip › plants-4348947-supplementary.pdf]

Supplementary material

Towards understanding the bioactive potential of two intriguing *Nepeta* species: metabolomic and phylogenetic perspectives

Uroš Gašić[1*], Tijana Banjanac[1], Luka Petrović[1], Jovana Petrović[1], Ladislav Luc[1], Branislav Šiler[1], Danijela Mišić[1], Milica Milutinović[1*]

[1] Department of Plant Physiology, Institute for Biological Research "Siniša Stanković" – National Institute of the Republic of Serbia, University of Belgrade, Bulevar despota Stefana 142, 11108 Belgrade, Serbia
* Correspondence: uros.gasic@ibiss.bg.ac.rs; milica.milutinovic@ibiss.bg.ac.rs

Figure S1. Heatmap of the scaled data of untargeted LC-MS metabolite analysis, with the samples (both columns and rows) arranged according to the HCA (Pearson method of cluster agglomeration). Intensity of green color indicate the abundance of the compounds in samples.

Figure S2. Relative abundances of antioxidative proteins. The obtained signal intensities for CAT, POX and PPO were normalized to the actin values, and the obtained results were normalized to the highest value and presented as relative abundances.

Table S1. Peak areas of the HPLC/Orbitrap MS data, corresponding to metabolites identified in methanol extracts *of N. subsessilis* (*NS*) and *N. govaniana* (*NG*) leaves.

Table S2. Peak areas of the metabolites identified in methanol extracts of *N. subsessilis* and *N. govaniana* leaves, as revealed by GC/MS analysis.

Table S3. GenBank accession numbers for DNA sequences used in this study to reconstruct phylogenetic relationships, as presented in Figure 3.

Table S4. Primer sequences and PCR conditions used for amplification of plastid loci.

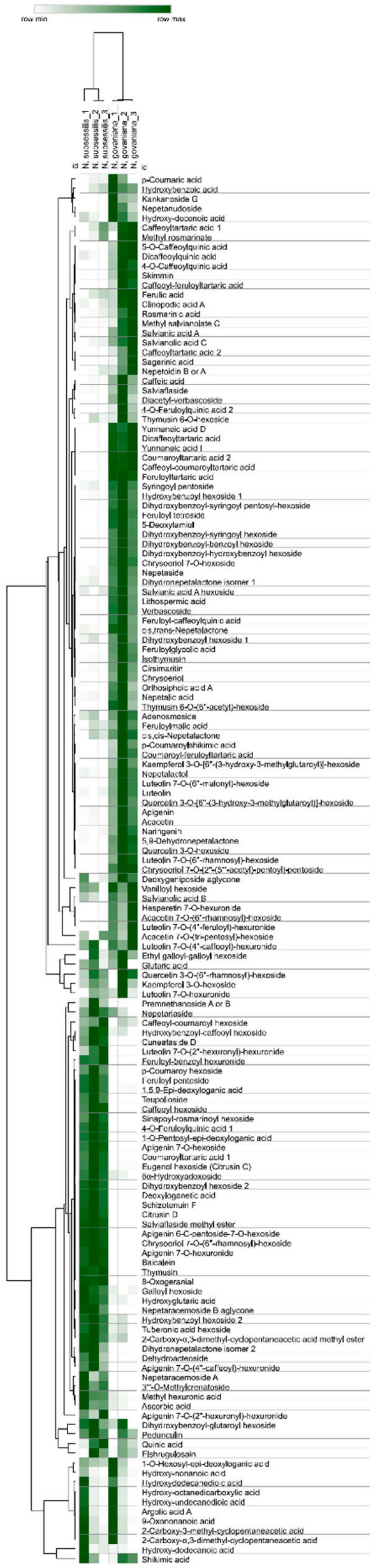
row min
row max
id
N. subsessilis_1
N. subsessilis_2
N. subsessilis_3
N. govaniana_1
N. govaniana_2
N. govaniana_3
id
p-Coumaric acid
Hydroxybenzoic acid
Kankanoside G
Nepetanudoside
Hydroxy-decenoic acid
Caffeoyltartaric acid 1
Methyl rosmarinate
5-O-Caffeoylquinic acid
Dicaffeoylquinic acid
4-O-Caffeoylquinic acid
Skimmin
Caffeoyl-feruloyltartaric acid
Ferulic acid
Clinopodic acid A
Rosmarinic acid
Methyl salvianolate C
Salvianic acid A
Salvianolic acid C
Caffeoyltartaric acid 2
Sagerinic acid
Nepetoidin B or A
Caffeic acid
Salviaflaside
Diacetyl-verbascoside
4-O-Feruloylquinic acid 2
Thymusin 6-O-hexoside
Yunnaneic acid D
Dicaffeoyltartaric acid
Yunnaneic acid I
Coumaroyltartaric acid 2
Caffeoyl-coumaroyltartaric acid
Feruloyltartaric acid
Syringoyl pentoside
Hydroxybenzoyl hexoside 1
Dihydroxybenzoyl-syringoyl pentosyl-hexoside
Feruloyl tetroside
5-Deoxylamiol
Dihydroxybenzoyl-syringoyl hexoside
Dihydroxybenzoyl-benzoyl hexoside
Dihydroxybenzoyl-hydroxybenzoyl hexoside
Chrysoeriol 7-O-hexoside
Nepetaside
Dihydronepetalactone isomer 1
Salvianic acid A hexoside
Lithospermic acid
Verbascoside
Feruloyl-caffeoylquinic acid
cis,trans-Nepetalactone
Dihydroxybenzoyl hexoside 1
Feruloylglycolic acid
Isothymusin
Cirsimaritin
Chrysoeriol
Orthosiphoic acid A
Nepetalic acid
Thymusin 6-O-(6"-acetyl)-hexoside
Adenosmoside
Feruloylmalic acid
cis,cis-Nepetalactone
p-Coumaroylshikimic acid
Coumaroyl-feruloyltartaric acid
Kaempferol 3-O-[6"-(3-hydroxy-3-methylglutaroyl)]-hexoside
Nepetalactol
Luteolin 7-O-(6"-malonyl)-hexoside
Luteolin
Quercetin 3-O-[6"-(3-hydroxy-3-methylglutaroyl)]-hexoside
Apigenin
Acacetin
Naringenin
5,9-Dehydronepetalactone
Quercetin 3-O-hexoside
Luteolin 7-O-(6"-rhamnosyl)-hexoside
Chrysoeriol 7-O-[2"-(5'"-acetyl)-pentoyl]-pentoside
Deoxygeniposide aglycone
Vanilloyl hexoside
Salvianolic acid B
Hesperetin 7-O-hexuronide
Acacetin 7-O-(6"-rhamnosyl)-hexoside
Luteolin 7-O-(4"-feruloyl)-hexuronide
Acacetin 7-O-(tri-pentosyl)-hexoside
Luteolin 7-O-(4"-caffeoyl)-hexuronide
Ethyl galloyl-galloyl hexoside
Glutaric acid
Quercetin 3-O-(6"-rhamnosyl)-hexoside
Kaempferol 3-O-hexoside
Luteolin 7-O-hexuronide
Premnethanoside A or B
Nepetariaside
Caffeoyl-coumaroyl hexoside
Hydroxybenzoyl-caffeoyl hexoside
Cuneataside D
Luteolin 7-O-(2"-hexuronyl)-hexuronide
Feruloyl-benzoyl hexuronide
p-Coumaroy hexoside
Feruloyl pentoside
1,5,9-Epi-deoxyloganic acid
Teupolioside
Caffeoyl hexoside
Sinapoyl-rosmarinoyl hexoside
4-O-Feruloylquinic acid 1
1-O-Pentosyl-epi-deoxyloganic acid
Apigenin 7-O-hexoside
Coumaroyltartaric acid 1
Eugenol hexoside (Citrusin C)
6α-Hydroxyadoxoside
Dihydroxybenzoyl hexoside 2
Deoxyloganetic acid
Schizotenuin F
Citrusin D
Salviaflaside methyl ester
Apigenin 6-C-pentoside-7-O-hexoside
Chrysoeriol 7-O-(6"-rhamnosyl)-hexoside
Apigenin 7-O-hexuronide
Baicalein
Thymusin
8-Oxogeranial
Galloyl hexoside
Hydroxyglutaric acid
Nepetaracemoside B aglycone
Hydroxybenzoyl hexoside 2
Tuberonic acid hexoside
2-Carboxy-α,3-dimethyl-cyclopentaneacetic acid methyl ester
Dihydronepetalactone isomer 2
Dehydroacteoside
Apigenin 7-O-(4"-caffeoyl)-hexuronide
Nepetaracemoside A
3'"-O-Methylcrenatoside
Methyl hexuronic acid
Ascorbic acid
Apigenin 7-O-(2"-hexuronyl)-hexuronide
Dihydroxybenzoyl-glutaroyl hexoside
Pedunculin
Quinic acid
Elshrugulosain
1-O-Hexosyl-epi-deoxyloganic acid
Hydroxy-nonanoic acid
Hydroxydodecanedioic acid
Hydroxy-octanedicarboxylic acid
Hydroxy-undecanedioic acid
Argolic acid A
9-Oxononanoic acid
2-Carboxy-3-methyl-cyclopentaneacetic acid
2-Carboxy-α,3-dimethyl-cyclopentaneacetic acid
Hydroxy-dodecanoic acid
Shikimic acid

Figure S1

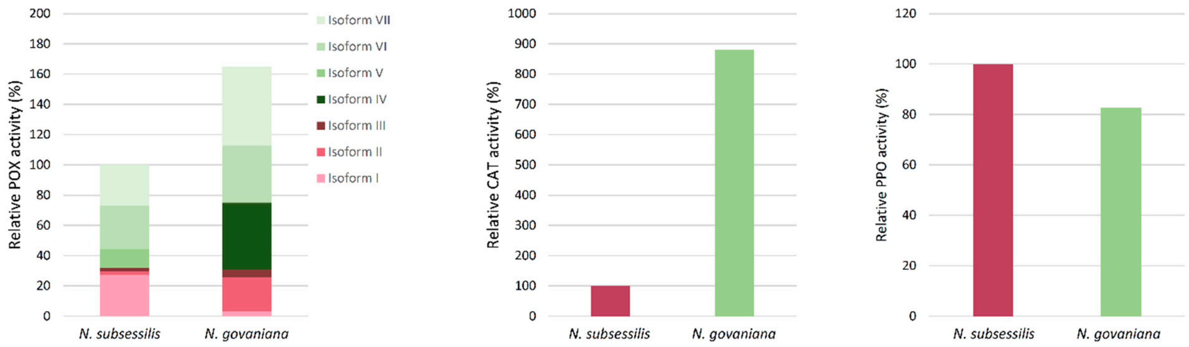

Relative POX activity (%)
Isoform VII
Isoform VI
Isoform V
Isoform IV
Isoform III
Isoform II
Isoform I
N. subsessilis
N. govaniana
Relative CAT activity (%)
N. subsessilis
N. govaniana
Relative PPO activity (%)
N. subsessilis
N. govaniana

Figure S2

Table S1

	*N. subsessilis*_1	*N. subsessilis*_2	*N. subsessilis*_3	*N. govaniana*_1	*N. govaniana*_2	*N. govaniana*_3
Galloyl hexoside	20124080	17823506	14975700	2661745	921670	1941546
Hydroxybenzoyl hexoside 1	1226560	1607408	1319472	18038871	22878460	15694673
Vanilloyl hexoside	2820261	2761510	2427012	3129100	2838280	3160405
Dihydroxybenzoyl hexoside 1	21351311	23212522	20089345	31502787	39289390	30362454
Syringoyl pentoside	759613	1004807	514418	5423327	6577054	4870101
Hydroxybenzoic acid	5245904	7431693	9425785	22584361	17031566	14497038
Dihydroxybenzoyl hexoside 2	2483731	2824585	2335445			
Dihydroxybenzoyl-glutaroyl hexoside	30845087	32932564	26865646	23455720	31994941	20655839
Hydroxybenzoyl hexoside 2	9124000	10055455	8239243	2559517	3297035	1821998
Dihydroxybenzoyl-syringoyl pentosyl-hexoside				18233938	23700804	16232207
Ethyl galloyl-galloyl hexoside	886712	1808418	986689	1400430	1956514	1347917
Dihydroxybenzoyl-syringoyl hexoside				1935094	2799640	1955919
Dihydroxybenzoyl-benzoyl hexoside				45254621	64028477	45460045
Dihydroxybenzoyl-hydroxybenzoyl hexoside				10372618	14247856	10015110
4-O-Feruloylquinic acid 1	2263634	2898135	2580885			
Caffeoyltartaric acid 1	2853320	7600738	22104902	10162692	33828456	36134778
Salvianic acid A hexoside	9090875	6066557	9721217	29173361	36340925	23039037
4-O-Caffeoylquinic acid				50052149	152295729	132393239
5-O-Caffeoylquinic acid				28071411	99167122	101450980
Coumaroyltartaric acid 1	26170494	36693302	31383708			
p-Coumaroy hexoside	6711476	11178769	7072640			
Caffeic acid	26036767	31314983	31973860	149265140	317658726	159846510
4-O-Feruloylquinic acid 2				131888	1263509	406676
Feruloylglycolic acid				987780	1618241	1036702
Lithospermic acid				3291726	4124291	2459160
p-Coumaric acid	196783	828722	811423	10861717	5510469	159221
Yunnaneic acid D				605631	540925	670337

Dicaffeoyltartaric acid				896152551	842490536	949814565
Feruloyl tetroside				1541493	1784707	1298280
Caffeoyltartaric acid 2				2721486	12025994	21330502
Feruloylmalic acid	1372114	2222462	1906302	3188122	5754044	4227187
Salviaflaside	431875	545228	318521	3303305	9757639	4107507
p-Coumaroylshikimic acid				604592	2533209	1512592
Salviaflaside methyl ester	927586	963522	891650			
Hydroxybenzoyl-caffeoyl hexoside	1451147	2628465	2495840	529950	707239	352660
Yunnaneic acid I				1508105	1406869	1609341
Dicaffeoylquinic acid	1206389	482248	166539	3556586	10829185	10969616
Salvianolic acid B	1353696	1095410	837124	2014798	1587897	2441699
Caffeoyl-feruloyltartaric acid				4241903	23547027	20365093
Feruloyl pentoside	3348867	4130014	3080107			
Caffeoyl hexoside	1832147	2644289	2502924			
Feruloyltartaric acid				4055997	4165540	3946455
Coumaroyltartaric acid 2				5775693	5610269	5941117
Caffeoyl-coumaroyltartaric acid				18936653	18542893	19330413
Ferulic acid	24368237	46618760	63365449	55961915	173370218	174360852
Rosmarinic acid	16668680	37878203	124153524	270040666	1061038011	1369041144
Salvianic acid A				2455028	4738345	5630106
Sagerinic acid				2207159	26382254	46968097
Caffeoyl-coumaroyl hexoside	1692857	1981664	2712126	994715	1463930	1229323
Skimmin				1884467	6242619	5804652
Coumaroyl-feruloyltartaric acid				736042	2678364	2023512
Feruloyl-caffeoylquinic acid				708549	926692	490406
Clinopodic acid A	2235385	1023684	3447086	4973836	18381201	19184580
Schizotenuin F	2936673	3290931	2740469			
Feruloyl-benzoyl hexuronide	375055	365005	469123			
Sinapoyl-rosmarinoyl hexoside	2080107	3238810	2683253			
Methyl rosmarinate	537126	764847	2352243	558548	3884267	3936765
Nepetoidin B or A	3746567	5794922	26355921	29862671	105107770	169117126

Salvianolic acid C	943184	3004221	4398522	20164283	34819224	43324692
Orthosiphoic acid A				340259	510835	169683
Methyl salvianolate C	904669	815729	993610	1692159	9096726	10563802
Teupolioside	5786832	8165233	5710435			
Dehydroacteoside	1135426	1565186	470479			
Verbascoside				1732221	2152455	1311987
3‴-O-Methylcrenatoside	1514953	842181	1178567			
Diacetyl-verbascoside				1825646	4201107	999414
Kankanoside G				4475771	1894803	1457561
Cuneataside D	666459	1223163	1100281			
Premnethanoside A or B	1443483	6497516	2062434			
1-O-Hexosyl-epi-deoxyloganic acid	12811112	10097153	8757655	22357267	9395390	4299391
Adenosmoside	47089083	54196962	39981205	66535556	87923549	77229552
Nepetanudoside				707624469	481707404	150209416
6α-Hydroxyadoxoside	28306460	34981941	29793592	9597548	7954611	6053191
1-O-Pentosyl-epi-deoxyloganic acid	3685836	4596392	4073151			
1,5,9-Epi-deoxyloganic acid	1951900841	2376632747	1777502674	35182468	66040453	92699531
5-Deoxylamiol				2483778	2961116	2006441
Nepetaracemoside A	2507446	659947	1583696			
Nepetaside	118234452	136887253	116978882	444654421	583839246	420534360
Nepetariaside	820409487	1219173134	1043623818	835015970	572572024	441658414
Deoxyloganetic acid	44647079	50750198	42352644			
Nepetaracemoside B aglycone	176608675	178666363	111819392			
Nepetalactol	14246888	13121380	11995871	30336073	48755709	39144427
Dihydronepetalactone isomer 1	125073495	138933492	112895842	311034511	408122200	292176350
Deoxygeniposide aglycone	2075170	384261	620722	1641102	2935067	2288085
Dihydronepetalactone isomer 2	15075776	15565592	6581795			
Nepetalic acid	22096698	23712289	15768662	177158177	239178676	108977106
5,9-Dehydronepetalactone	111951235	100383515	92742879	805204524	1344301812	1208930839
cis,trans-Nepetalactone	1752906114	782620398	497397677	18003462964	19831601151	11203670682
cis,cis-Nepetalactone	967648150	1382827069	1189324666	1855849876	2487988690	2237494127

Apigenin 6-C-pentoside-7-O-hexoside	4075825	4255476	3896173			
Luteolin 7-O-(2''-hexuronyl)-hexuronide	1133181	3461070	4520927			
Apigenin 7-O-(2''-hexuronyl)-hexuronide	1104728	449086	1760369			
Quercetin 3-O-(6''-rhamnosyl)-hexoside	2562967	3807039	3156056	1954436	4195357	3840297
Luteolin 7-O-(6''-rhamnosyl)-hexoside				909135	1980568	1795155
Quercetin 3-O-hexoside				4205472	7863719	6716842
Quercetin 3-O-[6''-(3-hydroxy-3-methylglutaroyl)]-hexoside	466591	580521	612225	9067791	19427074	12779473
Kaempferol 3-O-hexoside	9736246	15559749	12932738	5948632	21445649	10836931
Luteolin 7-O-hexuronide	21254940	26485262	25314539	23448561	32534002	22359382
Apigenin 7-O-hexoside	81468178	107549949	99727991			
Chrysoeriol 7-O-(6''-rhamnosyl)-hexoside	826118	861899	790337			
Apigenin 7-O-hexuronide	45140379	50227072	48735444	526699	668044	385353
Kaempferol 3-O-[6''-(3-hydroxy-3-methylglutaroyl)]-hexoside				2763216	7507891	4561706
Luteolin 7-O-(6''-malonyl)-hexoside				962166	2059291	1510729
Hesperetin 7-O-hexuronide				1347414	724233	1970595
Thymusin 6-O-hexoside	31301012	38111913	33169288	34863222	63261895	42834063
Luteolin 7-O-(4''-caffeoyl)-hexuronide	472226	756921	187531	668039	505880	830199
Acacetin 7-O-(tri-pentosyl)-hexoside	1624750	1320323	1929177	1971677	1603378	2339977
Acacetin 7-O-(6''-rhamnosyl)-hexoside				782825	409050	1156601
Thymusin 6-O-(6''-acetyl)-hexoside				4398645	4989067	1828990
Apigenin 7-O-(4''-caffeoyl)-hexuronide	609988	898178	321797			
Chrysoeriol 7-O-hexoside				10278391	13463094	10319162
Luteolin 7-O-(4''-feruloyl)-hexuronide				351422	173943	528902
Chrysoeriol 7-O-[2''-(5'''-acetyl)-pentoyl]-pentoside				1979106	2828393	2792838
Baicalein	1091892	1231745	1161818			
Thymusin	620285	595077	645494			
Luteolin	1173020	886595	1459446	5034488	9376034	7092331
Naringenin	717013	675575	758451	1634170	2926716	2458041
Apigenin	2608093	2716520	2548455	4693631	7518849	5900282
Isothymusin	26619607	36976306	33133719	320697999	544416667	380892854
Cirsimaritin	134838267	178279507	153687421	1499732195	2585821624	1492572289

Chrysoeriol	2127483	2535803	2394421	219196555	439827078	226490802
Pedunculin	3935638	5185636	4145838	1758656	4345988	2970208
Acacetin	19524913	31180125	25102161	206628148	441657251	311921876
Hydroxy-octanedicarboxylic acid	189060277	27435417	11533626	218980786	36447926	10324521
Hydroxy-undecanedioic acid	102543446	14171849	6346211	117856229	18163322	5214946
Hydroxy-decenoic acid	5093875	2982990	2157900	16298093	13094063	7078834
Hydroxydodecanedioic acid	6016035	937287	689595	4437211	976266	457770
9-Oxononanoic acid	12503187	2583018	782177	12618736	2701992	907498
Hydroxy-nonanoic acid	25746952	6657240	4116966	59540670	15303715	5713010
Hydroxy-dodecanoic acid	3467903	1014304	1188085	1016454	999869	882312
Methyl hexuronic acid	7387655	5536897	5396177	2109148	1755616	1501452
Shikimic acid	16065296	8612703	2238109	2958790	13186067	11304028
Quinic acid	209501738	274433223	294524006	201619634	246932148	220106414
Hydroxyglutaric acid	306510192	283644069	241621700	41448099	44042834	24978056
Glutaric acid	39331272	44321059	33278536	38870599	49536193	31968938
Ascorbic acid	19999881	11771659	12532137	3485785	2527538	1794621
Tuberonic acid hexoside	299773561	340636616	276251600	103420352	125487345	81353360
8-Oxogeranial	9519040	9358496	9679583			
Elshrugulosain	1150048	2594405	1921859	1180258	1889867	1564835
Citrusin D	2020450	2172681	1868218			
Eugenol hexoside (Citrusin C)	217448687	296867764	254589263	11221373	17444205	14017154
2-Carboxy-α,3-dimethyl-cyclopentaneacetic acid	68226926	20628847	12347418	69848754	13428802	5363900
2-Carboxy-α,3-dimethyl-cyclopentaneacetic acid methyl ester	56840945	59470854	47739183	23746436	28862082	17587482
Argolic acid A	455152542	71211774	34841046	493940386	92110661	27841303
2-Carboxy-3-methyl-cyclopentaneacetic acid	38948237	7909600	3403822	38693000	8165467	1703795

Table S2

	N. subsessilis 1	*N. subsessilis* 2	*N. subsessilis* 3	*N. govaniana* 1	*N. govaniana* 2	*N. govaniana* 3
a-Pinene	7080	6799	7959	4186	5207	6335
Sabinene	6284	5997	6690	4677	5147	5908
b-Pinene	11369	10698	12473	8396	8460	10104
b-Myrcene				5725	5584	5866
o-Cymene	4457	4474	4457			4610
D-Limonene	4599	4227	5121	4306	4185	4428
1,8-Cineole	47514	41451	39181	63769	45704	53785
d-Terpineol	2509	2248	1987	3715	2499	3027
Pulegone	6633	5418	4596	8194	5241	6437
trans,trans-Nepetalactone	1214169	1185209	936930	6797722	7814169	6477596
Unknow Nepetalactone Isomer	611770	648987	514147			
cis,trans-Nepetalactone	363126	376268	286320	1967247	2156882	1804239
trans,cis-Nepetalactone	92241	84490	58669			
cis,cis-Nepetalactone	57534	34639	25097	1345682	944272	496984
5,9-Dehydronepetalactone				97029	104049	111070
Dihydronepetalactone				10725	11232	10979
b-Caryoplhyllene	3557	3776	6528	48156	49510	35810
Humulene				35137	30046	22471
Germacrene D	13211	13451	10397	22842	18674	16214
γ-Himachalene				305962	299572	217506
Bicyclogermacrene				26219	20636	18154
Humulene epoxide I				8391	6907	4669
Neophytadiene 1	16392	20276	14307	13842	14616	9342
Phyt-2-ene	10582	14556	11296	8412	7379	7896
Neophytadiene 2	4398	4869	3927	3576	3692	3634
Neophytadiene 3	5101	5813	4209	4480	5739	2859
Phytol	268845	269304	143609	107048	82136	43579

Table S3

	Species	GenBank accessions:			DEFINITION
		matK	rbcL	trnLF	
1	***Nepeta laevigata* (D.Don) Hand.-Mazz.**	PQ870738.1			*Nepeta laevigata* maturase K (matK) gene, partial cds; plastid
			PQ767990.1		*Nepeta laevigata* ribulose-1,5-bisphosphate carboxylase-oxygenase
				PQ631085.1	*Nepeta laevigata* tRNA-Leu (trnL) gene and trnL-trnF intergenic spacer, partial sequence; chloroplast
2	***Nepeta grandiflora* M.Bieb.**	PQ870737.1			*Nepeta grandiflora* maturase K (matK) gene, partial cds; plastid
			PQ767989.1		*Nepeta grandiflora* ribulose-1,5-bisphosphate carboxylase-oxygenase large subunit (rbcL) gene, partial cds; chloroplast
				PQ631084.1	*Nepeta grandiflora* tRNA-Leu (trnL) gene and trnL-trnF intergenic spacer, partial sequence; chloroplast
3	***Nepeta rtanjensis* Diklić & Milojević**	PQ870742.1			*Nepeta rtanjensis* maturase K (matK) gene, partial cds; plastid
			PQ767994.1		*Nepeta rtanjensis* ribulose-1,5-bisphosphate carboxylase-oxygenase large subunit (rbcL) gene, partial cds; chloroplast
				PQ631089.1	*Nepeta rtanjensis* tRNA-Leu (trnL) gene and trnL-trnF intergenic spacer, partial sequence; chloroplast
4	***Nepeta ernesti-mayeri* Diklić & V.Nikolić**	PQ870736.1			*Nepeta ernesti-mayeri* maturase K (matK) gene, partial cds; plastid
			PQ767988.1		*Nepeta ernesti-mayeri* ribulose-1,5-bisphosphate carboxylase-oxygenase large subunit (rbcL) gene, partial cds; chloroplast.
				PQ631083.1	*Nepeta ernesti-mayeri* tRNA-Leu (trnL) gene and trnL-trnF intergenic spacer, partial sequence; chloroplast
5	***Nepeta cataria* L.**	PQ870735.1			*Nepeta cataria* maturase K (matK) gene, partial cds; plastid
			PQ767987.1		*Nepeta cataria* ribulose-1,5-bisphosphate carboxylase-oxygenase large subunit (rbcL) gene, partial cds; chloroplast
				PQ626047.1	*Nepeta cataria* tRNA-Leu (trnL) gene and trnL-trnF intergenic spacer, partial sequence; chloroplast
6	***Nepeta parnassica* Heldr. & Sartori**	PQ870741.1			*Nepeta parnassica* maturase K (matK) gene, partial cds; plastid
			PQ767993.1		*Nepeta parnassica* ribulose-1,5-bisphosphate carboxylase-oxygenase large subunit (rbcL) gene, partial cds; chloroplast

				PQ631088.1	*Nepeta parnassica* tRNA-Leu (trnL) gene and trnL-trnF intergenic sspacer, partial sequence; chloroplast
7	***Nepeta ucranica* L.**	PQ348678.1			*Nepeta ucranica* voucher KG24-0093 maturase K (matK) gene, partial cds; chloroplast
			PQ337439.1		*Nepeta ucranica* voucher KG24-0093 ribulose-1,5-bisphosphate carboxylase/oxygenase large subunit (rbcL) gene, partial cds; chloroplast
				OR199264	*Nepeta ucranica* isolate A. Naumenko s.n. (NEBK) trnL-trnF intergenic spacer region, partial sequence; plastid
8	***Nepeta nuda* L.**	PQ870740.1			*Nepeta nuda* maturase K (matK) gene, partial cds; plastid
			PQ767992.1		*Nepeta nuda* ribulose-1,5-bisphosphate carboxylase-oxygenase large subunit (rbcL) gene, partial cds; chloroplast
				PQ631087.1	*Nepeta nuda* tRNA-Leu (trnL) gene and trnL-trnF intergenic spacer, partial sequence; chloroplast
9	***Nepeta nervosa* Royle ex Benth.**	PQ870739.1			*Nepeta nervosa* maturase K (matK) gene, partial cds; plastid
			PQ767991.1		*Nepeta nervosa* ribulose-1,5-bisphosphate carboxylase-oxygenase large subunit gene, partial cds; chloroplast
				PQ631086.1	*Nepeta nervosa* tRNA-Leu (trnL) gene and trnL-trnF intergenic spacer, partial sequence; chloroplast
10	***Nepeta sibirica* L.**	PQ870743.1			*Nepeta sibirica* maturase K (matK) gene, partial cds; plastid
			PQ767995.1		*Nepeta sibirica* ribulose-1,5-bisphosphate carboxylase-oxygenase large subunit (rbcL) gene, partial cds; chloroplast
				PQ631090.1	*Nepeta sibirica* tRNA-Leu (trnL) gene and trnL-trnF intergenic spacer, partial sequence; chloroplast
11	***Nepeta govaniana* (Wall. ex Benth.) Benth.**	Z349586			*Nepeta govaniana* maturase K (matK) gene, partial cds; plastid
			Z349587		*Nepeta govaniana* ribulose-1,5-bisphosphate carboxylase-oxygenase large subunit (rbcL) gene, partial cds; chloroplast
				Z349588	*Nepeta govaniana* tRNA-Leu (trnL) gene and trnL-trnF intergenic spacer, partial sequence; chloroplast
12	***Nepeta subsessilis* Maxim.**	Z349589			*Nepeta subsessilis* maturase K (matK) gene, partial cds; plastid
			Z349590		*Nepeta subsessilis* ribulose-1,5-bisphosphate carboxylase-oxygenase large subunit (rbcL) gene, partial cds; chloroplast

		Z349591	*Nepeta subsessilis* RNA-Leu (trnL) gene and trnL-trnF intergenic spacer, partial sequence; chloroplast
13	***Nepeta olgae* Regel**	Nikitina, E., and Rakhmatov, A. (2021). Identification of *Nepeta olgae* Regel and phylogenetic status of some genera in subtribe Nepetinae (Lamiaceae) using DNA markers, in BIO Web of Conferences, (EDP Sciences), 00087. Available at: https://www.bio-conferences.org/articles/bioconf/abs/2021/10/bioconf_napd2021_00087/bioconf_napd2021_00087.html (Accessed December 6, 2023).	
14	***Nepeta bracteata* Benth.**	NC_079688.1	*Nepeta bracteata* chloroplast, complete genome
15	***Nepeta coerulescens* Maxim. (syn. *N. thomsonii*)**	NC_071850.1	*Nepeta thomsonii* voucher H3050023 chloroplast, complete genome
16	***Nepeta stewartiana* Diels**	NC_057283.1	*Nepeta stewartiana* isolate BOP018025 chloroplast, complete genome
17	***Nepeta hemsleyana* Oliv. ex**	NC_058882.1	*Nepeta hemsleyana* chloroplast, complete genome
18	***Nepeta santoana* Popov**	PV837617.1	*Nepeta santoana* voucher TASH072436 chloroplast, complete genome
19	***Nepeta apuleji* Ucria (syn. *N. dentata*)**	NC_071848.1	*Nepeta dentata* voucher H3050533 chloroplast, complete genome
20	***Dracocephalum ruyschiana***	PQ963003.1	*Dracocephalum ruyschiana* voucher UBU0038422 chloroplast, complete genome
21	***Nepeta tenuifolia* Benth.**	NC_061322.1	*Nepeta tenuifolia* chloroplast, complete genome
22	***Salvia aerea***	NC_067739.1	*Salvia aerea* voucher GX Hu 614 chloroplast, complete genome
23	***Lavandula angustifolia***	NC_029370.1	*Lavandula angustifolia* chloroplast, complete genome
24	***Origanum vulgare* subsp. *vulgare***	JX880022.1	*Origanum vulgare* subsp. *vulgare* chloroplast, complete genome
25	***Mentha arvensis***	PX971240.1	*Mentha arvensis* chloroplast, complete genome
26	***Lamium album***	OR565909.1	*Lamium album* voucher ZY013 chloroplast, complete genome

Table S4

Plastid region	Primers	Primer sequences (5′- 3′)	PCR conditions	
***mat*K**	matK_390F	CGATCTATTCATTCAATATTTC	94◦C 4 min<br>31× 94◦C 30 s<br>51◦C 60 s<br>72◦C 1 min,	Cheng et al. 2015
	matK_1326R	CGATCTATTCATTCAATATTTC	72◦C 4 min	
***rbc*L**	rbcLa_F	ATGTCACCACAAACAGAGACTAAAGC	94◦C 10 min<br>25× 94◦C 1 min<br>55.1◦C 45 s<br>72◦C 90 s	Avanesyan, Sutton, and Lamp 2021
	rbcLa_R	GTAAAATCAAGTCCACCRCG	72◦C 10 min	
***trn*L-F**	c	CGAAATCGGTAGACGCTACG	94◦C 10 min<br>35× 94◦C 1 min<br>55◦C 45 s<br>72◦C 90 s	Taberlet et al. 1991
	f	ATTTGAACTGGTGACACGAG	72◦C 10 min	
